# From Small Data Modeling to Large Language Model Screening: A Dual‐Strategy Framework for Materials Intelligent Design

**DOI:** 10.1002/advs.202403548

**Published:** 2024-10-04

**Authors:** Yeyong Yu, Jie Xiong, Xing Wu, Quan Qian

**Affiliations:** ^1^ School of Computer Engineering & Science Shanghai University Shanghai 200444 China; ^2^ Center of Materials Informatics and Data Science, Materials Genome Institute Shanghai University Shanghai 200444 China; ^3^ Key Laboratory of Silicate Cultural Relics Conservation (Shanghai University) Ministry of Education Shanghai 200444 China; ^4^ Shanghai Institute for Advanced Communication and Data Science Shanghai University Shanghai 200444 China

**Keywords:** adversarial domain adaptation, experimental candidates screening, material intelligent design, small data modeling

## Abstract

Small data in materials present significant challenges to constructing highly accurate machine learning models, severely hindering the widespread implementation of data‐driven materials intelligent design. In this study, the Dual‐Strategy Materials Intelligent Design Framework (DSMID) is introduced, which integrates two innovative methods. The Adversarial domain Adaptive Embedding Generative network (AAEG) transfers data between related property datasets, even with only 90 data points, enhancing material composition characterization and improving property prediction. Additionally, to address the challenge of screening and evaluating numerous alloy designs, the Automated Material Screening and Evaluation Pipeline (AMSEP) is implemented. This pipeline utilizes large language models with extensive domain knowledge to efficiently identify promising experimental candidates through self‐retrieval and self‐summarization. Experimental findings demonstrate that this approach effectively identifies and prepares new eutectic High Entropy Alloy (EHEA), notably Al_14_(CoCrFe)_19_Ni_28_, achieving an ultimate tensile strength of 1085 MPa and 24% elongation without heat treatment or extra processing. This demonstrates significantly greater plasticity and equivalent strength compared to the typical as‐cast eutectic HEA AlCoCrFeNi_2.1_. The DSMID framework, combining AAEG and AMSEP, addresses the challenges of small data modeling and extensive candidate screening, contributing to cost reduction and enhanced efficiency of material design. This framework offers a promising avenue for intelligent material design, particularly in scenarios constrained by limited data availability.

## Graphical abstract

The Dual‐Strategy Materials Intelligent Design Framework (DSMID) tackles the challenges of small data in materials intelligent design. By enhancing property prediction and efficiently screening experimental candidates, DSMID successfully identifies a new eutectic High Entropy Alloy with significantly improved plasticity and strength, providing a powerful solution for material intelligent design in data‐constrained scenarios.

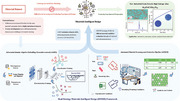

## 1 Introduction

The innovative design and development of new materials play a critical role in advancing technology across various sectors, including energy,^[^ ^1^ ^]^ electronics,^[^ ^2^ ^]^ healthcare,^[^ ^3^ ^]^ etc. Yet, the field of materials science encounters a significant barrier that hinders the swift progression of these innovations: the limitations imposed by small sample datasets.^[^ ^4^, ^5^ ^]^ Such datasets restrict the accuracy and reliability of machine learning (ML) models designed to predict material properties and behaviors,^[^ ^6^ ^]^ essential for identifying materials with desired features. This data scarcity bottleneck not only undermines the effectiveness of predictive models but also presents a considerable challenge to the innovative design of materials. Despite the advances in machine learning techniques, the scarcity of large, labeled datasets in materials science remains a critical obstacle.

To address this challenge, integrating machine learning with techniques such as transfer learning, as detailed in Section S1 (Supporting Information), can effectively mitigate data scarcity in materials science. Transfer learning methods, including feature transfer, parameter transfer, and sample transfer,^[^ ^7^ ^]^ can leverage knowledge from related fields to enhance predictive models. Among these, feature transfer through domain adaptation is particularly useful. It addresses domain shift–common in material datasets due to varying origins, preparation procedures, and experimental conditions–by aligning characteristics across different data sources, thereby reducing biases and improving the accuracy of material property predictions.

Deep neural network‐based domain adaptation algorithms have shown great potential in this context. Techniques such as Deep Adaptation Network (DAN),^[^ ^8^ ^]^ Domain Adversarial Neural Network (DANN),^[^ ^9^ ^]^ and Conditional Adversarial Domain Adaptation (CDAN),^[^ ^10^ ^]^ are all unsupervised, aggregating only source domain information to achieve effective transferability to the target domain. Conversely, adversarial domain adaptation algorithms^[^ ^11^ ^]^ require a large amount of image data to converge. However, some challenges exist when using these algorithms to process small sample dataset of materials, which include:

1) Due to measurement difficulties, limited experimental data, and insufficient data volume compared to other relevant datasets, certain properties of materials have lower predictive accuracy.
2) Current domain adaptation methods are unsupervised in the target domain. Without considering the correlation between target and source domain performance, it is challenging to achieve good results through feature transfer between different materials performance datasets using unsupervised domain adaptation methods.
3) Domain knowledge‐based connections exist among several performance characteristics of materials. Integrating such knowledge into domain adaptation networks can significantly improve the models' interpretability and generalization ability.
4) Domain adaptation techniques, based on Generative Adversarial Networks (GANs), are incapable of producing authentic images with minimal materials datasets. Likewise, pixel‐based domain adaptation methods encounter challenges when it comes to convergence.

To address the above‐mentioned challenges, we innovated in adversarial domain adaptation by incorporating domain knowledge into the training process, thus strengthening the connection between source and target datasets beyond the reliance on GAN‐based image generation. Our methodology focused on refining the feature representation network through the use of detailed gradient information, employing Correlation Alignment (CORAL)‐based loss, residual connections, and gradient reversal layers to mitigate domain shift. This approach was designed to obtain improved representation embeddings for material components and develop a precise performance predictor.

Expanding the discussion on advancing intelligent material design with our innovative method, it is essential to apply these concepts to specific materials like High Entropy Alloys (HEAs).^[^ ^12^ ^]^ The exceptional properties of HEAs, including enhanced specific strength, fracture resistance, and corrosion resistance,^[^ ^13^ ^]^ along with their wide‐ranging industrial applications from aerospace^[^ ^14^ ^]^ to electronics,^[^ ^15^ ^]^ highlight the necessity of reverse engineering HEAs.

The diversity and performance of HEAs^[^ ^12^, ^13^ ^]^ are primarily determined by their multi‐component compositions, driving research toward optimizing alloy organization through element type and content control. However, the traditional experimental approach to alloy design is resource‐intensive, underscoring the critical need to develop precise predictive models and streamline the candidate evaluation pipeline for the rapid design of HEAs, thus reducing development costs and time.

Motivated by these factors, we investigate the application of domain adaptation methodologies in the HEAs characterization and design field:

1) The mechanical properties of HEAs, including their elastic and plastic behaviors, hardening mechanisms, and creep behavior, are diverse.^[^ ^16^, ^17^ ^]^ The hardness (HV) test is favored for its simplicity, minimal sample preparation, quick and accurate results, and high precision. However, ultimate tensile strength (UTS) and elongation (EL) tests are more challenging due to the brittleness of HEAs, which complicates standard sample creation and limits data availability for precise modeling.
2) Empirical evidence indicates a correlation between hardness and plastic strength in metallic materials, where higher material strength corresponds to greater resistance to plastic deformation, resulting in higher hardness.^[^ ^18^, ^19^, ^20^ ^]^ Consequently, applying domain adaptation to various properties within the same HEAs is both a natural and knowledge‐supported approach.

In this study, we propose an **Adversarial domain Adaptive Embedding Generative network (AAEG)** aimed at transferring predictive tasks for different properties of the same material. Leveraging a dataset of 290 accurately measured HV as the source domain, we transferred knowledge to predict UTS and EL in the target domains, which are challenging to measure and consist of only 90 samples. Our method significantly improves the prediction accuracy for HV in the source domain, as well as UTS and EL in the target domains, demonstrating its efficacy and potential for broader applications in material property prediction.

Establishing a high‐precision predictive model is essential for supporting and ensuring the reliability of reverse materials design. However, such a model alone cannot facilitate experimental validation, which significantly weakens our impact in intelligent materials design. By leveraging a high‐precision model established through transfer learning, we employed the Non‐dominated Sorting Genetic Algorithm II (NSGA‐II)^[^ ^21^ ^]^ algorithm to generate a diverse range of initial material preparation candidates and predict their potential performance. However, preparing all the proposed candidates is impractical due to time and cost constraints. Thus, it is crucial for material experts to screen these candidates, using their expertise to eliminate obviously unfeasible options and select the most promising experimental candidates.

To address the challenges associated with the high threshold and long duration of manual candidate selection, we leveraged the capabilities of large language models (LLMs).^[^ ^22^ ^]^ These models possess extensive domain knowledge and can efficiently screen and evaluate numerous alloy designs, significantly streamlining the selection pipeline.

We introduced an **Automated Material Screening and Evaluation Pipeline (AMSEP)** wherein LLMs perform self‐retrieval and self‐summarization to efficiently identify the most promising high‐entropy alloys. This automated approach not only enhances screening efficiency but also ensures the accuracy and reliability of the results. By leveraging the advanced capabilities of LLMs, our method accelerates the discovery and development of new materials, representing a significant breakthrough in materials science research.

Leveraging the integration of AAEG and AMSEP, we developed the **Dual‐Strategy Materials Intelligent Design (DSMID) Framework**. This framework capitalizes on AAEG's predictive prowess for hard‐to‐measure properties and AMSEP's efficient candidate screening. DSMID effectively addresses small sample size challenges by merging transfer learning and AI‐driven evaluations, accelerating the material design process. This strategic combination enables the rapid development of materials that meet specific performance requirements.

**Figure 1:**
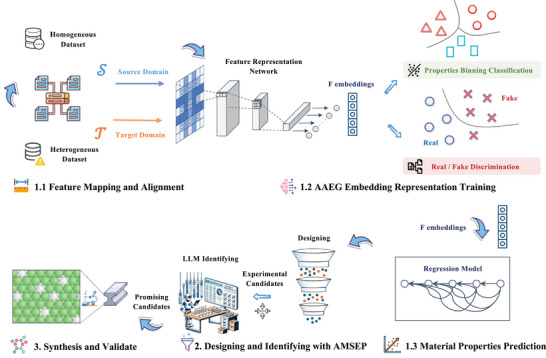
Overview of the **Dual‐Strategy Materials Intelligent Design (DSMID) Framework**. **Adversarial domain Adaptive Embedding Generative network (AAEG)** enhances material property prediction by aligning feature representations across domains using domain‐adaptive techniques. **Automated Material Screening and Evaluation Pipeline (AMSEP)** utilizes NSGA‐II and large language models to generate and identify materials with superior mechanical properties. **Synthesis and Validate** confirms the model's predictive accuracy and supports the reverse design of materials.

The DSMID Framework integration expedited the reverse engineering process for intelligent material design, as shown in **Figure** **1**. The following detailed breakdown elaborates on the key components and their functionalities:

1) **AAEG for Material Properties Prediction**: We developed an AAEG to align the feature representations of different material properties, significantly enhancing the accuracy of property predictions.
  1.1 **Feature Mapping and Alignment**: The source and target domains belong to the same type of materials, but the data features are misaligned due to their different sources. To align the features, it is necessary to map the essential component features of the materials to the same space using Xenonpy,^[^ ^23^ ^]^ and then perform feature alignment.
  1.2 **AAEG Embedding Representation Training**: We propose a domain‐adaptive method, which induces a symbiotic relationship between learning embeddings and generative adversarial networks to make the joint feature space closer to the source and target distributions.
  1.3 **Material Properties Prediction**: With AAEG, we established high‐precision predictors for material performance and participated in reverse design processes.
2) **Designing and Identifying with AMSEP**: By employing the LLMs, we meticulously evaluated candidates from the NSGA‐II algorithm, identifying materials with outstanding mechanical properties.
3) **Synthesis and Validate**: Finally, the predicted accuracy and extrapolation ability of our model were verified through the experimental preparation of materials, providing significant theoretical guidance for reverse material design.

Building on this foundation, our DSMID Framework has led to the creation of a novel eutectic HEA Al_14_(CoCrFe)_19_Ni_28_ within our established dataset. This HEA aligns with eco‐friendly manufacturing principles and demonstrates significantly greater plasticity without strength loss compared to the typical as‐cast eutectic HEA AlCoCrFeNi_2.1_.^[^ ^24^ ^]^

The primary contributions of the paper can be summarized as follows:

1) **Advanced Material Characterization**: We developed an AAEG network employing adversarial generative techniques and coupled feature representation to minimize domain shifts across varied material performance datasets, improving accuracy in property predictions under constrained data conditions.
2) **Efficient Candidates Identification**: Addressing the challenge of evaluating numerous material candidates, we introduced the AMSEP process, which utilizes LLMs for efficient identification through self‐retrieval and self‐summarization, leveraging extensive domain knowledge.
3) **Integrated DSMID Framework**: We introduced the DSMID framework, integrating AAEG and AMSEP to enhance prediction accuracy and streamline the discovery and evaluation of new materials, significantly accelerating intelligent design.
4) **Industrial‐Grade EHEA Development**: Through the DSMID framework, we developed an industrial‐grade EHEA that meets green manufacturing standards. This alloy, requiring no additional processing, achieves a yield strength of 362 MPa, ultimate tensile strength of 1085 MPa, and elongation at break of 23.8%, marking a significant advance in HEA development.

The structure of this paper is as follows: Section 2 provides a detailed introduction to the proposed DSMID framework and its specific outcomes, including the application and performance of the AAEG and AMSEP methods. Section 3 summarizes the main findings of the study and proposes directions for future research. Section 4 supplements with implementation details of the DSMID framework and additional experimental results.

## 2 Results

### 2.1 Dual‐Strategy Materials Intelligent Design Framework

#### 2.1.1 Adversarial Domain Adaptive Embedding Generative Network

To fully utilize the experimental data of the high‐entropy alloy HV target performance to help establish a model for predicting the UTS and EL target performance, it is imperative to align the characteristics of the domains. Due to differences in constituent components in the two datasets, we employ a method^[^ ^25^ ^]^ to expand the composition through XenonPy. This method calculates atomic features using the seven statistical formulas provided in Table S5 (Supporting Information). Subsequently, these calculated statistical features are transformed into 2D images, as shown in Figure S2 (Supporting Information). As these features are statistical, they are significantly denser compared to many zero‐valued component features.

Traditional transfer learning methods, constrained by the small‐sample problem in materials and lacking utilization of connections between different properties of the same material, perform domain adaptation on feature‐aligned images. So we propose a GAN‐based method to bridge the domain gap between the source and target domains. The neural network is employed to extract features using a generative and discriminative process in the adversarial, ensuring the transfer of rich information from the source and target domains to the learned embeddings. Crucially, this approach does not rely on the successful pixel‐level generation of images in an adversarial generation. Instead, it leverages GANs and binning tasks to provide effective transfer learning gradients for our material composition representation networks, demonstrating robust domain adaptation capabilities even with small material samples.

Through adversarial transfer learning, the neural network identifies potential correlations between source and target domain component features, subsequently generating more accurate and effective machine learning models based on these extracted features.

**Symbol Definitions**. In this Part, we will introduce some symbols and definitions used in the AAEG.

1) D={X,P(X)} represents the domain, which consists of two parts: the feature space X and the marginal probability distribution *P*(*X*), where $X=\{x_{1},\text{…},x_{n}\}\in X$.^[^ ^26^ ^]^
2) The source domain is denoted as S, with $X_{S}$ represents the feature space of the source domain, and $X_{S}=\left\{x_{S1},\text{…},x_{Sn}\right\}\in X_{S}$ where $X_{S}$ represents the component feature image input of the source domain. T represents the target domain, $X_{T}$ represents the feature space of the target domain, and $X_{T}$ represents the component feature image input of the target domain.
3) Since GAN networks (as described in Section S4.2.1, Supporting Information) are primarily used for image classification and often fail to converge on regression problems, we categorize the performance of the HV target into separate bins, where $L=\{0,\;1,\;2\text{…}N_{c}\}$ and *N*_*c*_ is the number of bins.
4) In the regression problem, *Y*_*HV*_ = {*y*_*i*_}*i* = 1^*N*^ represents the measured actual values of HV in the source domain, *Y*_*UTS*_ = {*y*_*i*_}*i* = 1^*N*^ and $Y_{EL}={\left\{y_{i}\right\}}_{i=1}^{N}$ represent the measured actual values of UTS and EL, respectively, in the target domain. *Y*_*c*_ = {*Y*_*T*_, *Y*_*F*_} represents the true or false labels of the image, where *Y*_*T*_ represents the real image and *Y*_*F*_ represents the generated image.

Notation commonly used throughout this paper is documented in Table S3 (Supporting Information). The correspondence between abbreviations and their full terms can be found in Table S4 (Supporting Information).

**Figure 2:**
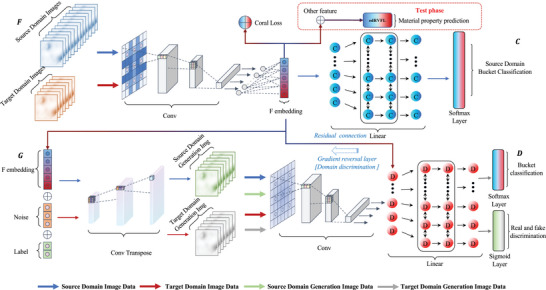
Adversarial domain adaptive embedding generative network structure. F: Feature representation network, whose output of the last hidden layer is used as the generated embedding of materials composition. C: Source domain bucketing network, used to provide gradients for F to perform source domain bucketing task. G: Generation network, used to provide gradients for F to confuse the source and target domain. D: Discriminator network is designed to perform two critical tasks: discriminate between the source and target domains for classification and source domain target properties bucketing. The network is adversarially trained with G and F and is directly transferred to F utilizing residual connections to minimize domain shift between the source and target domains.

**Establishing AAEG Model**. The network architecture is divided into four separate networks, as illustrated in **Figure** **2**.

- **Feature representation network *F***: $X\mapsto R^{d}$. The network *F* implicitly learns the domain shift between S and T by extracting information from the target data during training.
- **Source domain bucketing network *C***: $R^{d}\mapsto L$. The network *C* can access labels of data sampled from the source distribution but lacks access to those from the target distribution.
- **Generator network *G***: 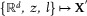. The network *G* assists in generating images for adversarial training in conjunction with the *D*, utilizing the feature representation output provided by the *F*. Throughout the adversarial process, it aids the *F* in enhancing the performance of component feature representation.
- **Discriminator network *D***: 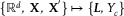. The network *D* facilitates gradient propagation training of the *F* by engaging in adversarial interactions with the *G* and utilizing residual connections and gradient reversal layers between the *F*.

Adversarial training is adopted by both networks to support *F* in extracting more potent embedding vectors for the ultimate regression tasks.

**Overview of AAEG Networks**. The inputs to the four distinct networks of the AAEG model and their respective training tasks are described in the following part, as detailed in Figure 2.

As material datasets are generally limited in scale due to the high cost and lengthy periods required for experimentation, achieving the convergence of GANs typically necessitates a significant amount of data. Because the primary objective of this study is to enhance the regression prediction accuracy of material properties in the target domain, the objective of the AAEG is to generate a universal material composition representation embedding by learning from both the source and target domains. This representation should then be evaluated in various downstream material characteristic prediction tasks, to establish a more precise regression predictor subsequently.

Furthermore, the primary objective of the generator network shifts from generating realistic composition feature images to providing gradients that decrease domain shift to the component representation network F. As regression problems present challenges within domain adaptation theory and applications, we discretize the regression task into binning labels for HV target performance, with both C and D predicting these bucketing labels. The inputs, outputs, and training tasks of the network, interconnected by *F*, *C*, *G*, and *D*, are detailed through the following steps:

1) **Embedding Extraction and Binning Classification**: The real images from the $X_{S}$ are fed into F to obtain their corresponding embeddings represented by *F*(*x*). Subsequently, C is used to predict the probability distribution of HV binning through the input of *F*(*x*), represented as *C*(*F*(*x*)).
2) **Image Generation with Conditional Inputs**: The generator network G takes an input *x*_*g*_ = [*F*(*x*), *z*, *l*], where *z* is a noise vector of dimension *d* that follows the normal distribution of N(0,1), and *l* is a one‐hot vector obtained through the binning labels denoted by L with dimension *N*_*c*_ + 1. This dimension is used to indicate the source of *F*(*x*), where 0 denotes the source domain, and 1 denotes the target domain. *G* generates images *G*(*x*_*g*_) as the output.
3) **Discriminator Network Functionality**: The discriminator network *D* has two inputs: one is the generated images *G*(*x*_*g*_) from *G*, and the other is the real images *x*. The input also includes a residual connection embedding *F*(*x*). It has two outputs:
  ① *D*_*data*_(*x*), which models the probability of input *x* being a real image from the source domain and acts as a binary classifier.
  ② *D*_*cls*_(*x*), which outputs the probability distribution of binning labels for input *x* and matches the output of *C* network.
  As generated images lack binning labels, only *D*_*data*_(*x*_*g*_) can back‐propagate gradients. Unlike ref. [11], since binning labels also exist in the target domain, $D_{cls}\left(x_{T}\right)$ of the target domain can back‐propagate gradients as well.
4) **Adversarial Gradient Feedback to** ***F***: We introduce a residual connection between *F* and *D* to allow for the gradients from the fully connected layer on the latter half of *D* to back‐propagate to *F*. Notably, It is important to note that *F* and *D* engage in an adversarial relationship, where *F* aims to generate $F(x_{T})$ that *D* classifies as originating from the source domain, whereas *D* aims to classify it as not from the source domain. When back‐propagating gradients, the Gradient Reversal Layer (GRL, as described in Section S4.2.3, Supporting Information) is applied to reverse gradients of $D_{data}\left(F\left(x_{T}\right)\right)$ as it propagates, while $D_{data}\left(F\left(x_{S}\right)\right)$ and *D*_*cls*_(*F*(*x*)) back‐propagate normally.

In Section 4.1, we provide a detailed description of the iterative training process for AAEG, which is summarized by the pseudocode in **Algorithm** **1**. However, due to issues with convergence in regression tasks for domain adaptation, we adopt a coarse‐grained bucketing approach to closely examine HV target performance, resulting in the decomposition of regression tasks into classification tasks. This technique assures ***F*** **is trained on plentiful data from the source domain while boosting the generalization capacity of its embedding**. The inclusion of adversarial transfer and CORAL loss (as described in Section S4.2.2, Supporting Information) assists in creating target domain embeddings that comply with the source domain's distribution and minimize domain shift. The ultimate hidden state of ***F*** **is employed to encode high‐entropy ingredient information of a given alloy recipe** and concatenated with heat treatment features. Such modifications allow for improved regression model training in the target domain, leading to better embedding representation and increased accuracy. The mutual relationship among Embedding, Adversarial Transfer framework, and CORAL Loss plays a crucial role in the proposed method's success.

Overall, the AAEG framework, with its innovative use of adversarial learning and coarse‐grained bucketing strategies, not only overcomes the challenges of small sample sizes in material domains but also harnesses the nuanced inter‐property relationships within materials. This enables more effective domain adaptation and enhanced predictive performance. Detailed training and testing processes for AAEG can be found in Algorithm S1 (Supporting Information). The parameters of the AAEG networks can be found in Table S7 (Supporting Information), and specific experimental outcomes are illustrated in Section 2.3.1.

#### 2.1.2 Automated Material Screening and Evaluation Pipeline

To tackle the challenge of screening and evaluating numerous alloy designs, we introduced an automated pipeline utilizing large language models. This section details the implementation and efficacy of this innovative approach.

The NSGA‐II is a widely used genetic algorithm for multi‐objective optimization, known for maintaining solution diversity through non‐dominated sorting and crowding distance comparison.^[^ ^21^ ^]^ In the realm of materials reverse design, NSGA‐II is employed to optimize material properties. By incorporating the high‐precision performance prediction capabilities of the AAEG, NSGA‐II can efficiently and accurately identify experimental candidates that satisfy multiple performance criteria. These experimental candidates include the composition, preparation process, and AAEG‐predicted properties of novel HEAs, including HV, UTS, and EL.

**Figure 3:**
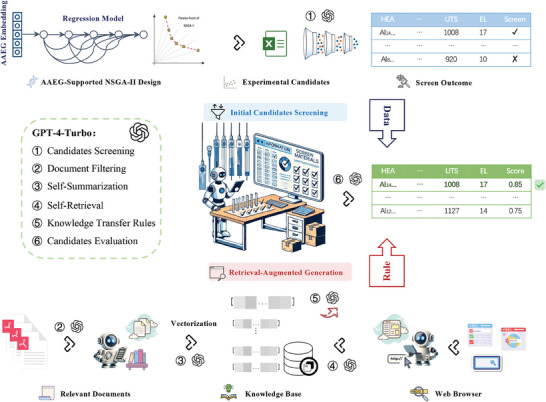
Automated Screening and Evaluation of Inverse Design Candidates via Joint Data and Rule Stream Approach.

Traditional experimental candidates screening heavily relies on expert knowledge and is time‐consuming. To expedite and streamline the identification of the most promising research and application experimental candidates, we have developed and implemented a series of automated procedures for selecting and evaluating experimental candidates, as illustrated in **Figure** **3**. These automated procedures have significantly optimized the complex decision‐making process in candidate selection, thereby enhancing both the efficiency and accuracy of the selection process. Details of the specific automated pipeline are provided below:

1) **Camdidates Screening**: We conducted an initial screening of the extensive array of experimental candidates, derived through reverse design via the NSGA‐II algorithm, utilizing LLM. This phase entails a thorough examination of essential candidates, including the target system, required properties, and processing limitations, to isolate candidates that satisfy established criteria.
2) **Document Filtering**: To diminish the expenses associated with creating a vector knowledge query library, LLM selectively identifies pertinent literature by analyzing titles and abstracts in alignment with the task description.
3) **Self‐Summarization**: Following a self‐summarization of the curated literature with LLM, this refined content underpins the subsequent process of document segmentation and construction of a vectorized knowledge base.
4) **Retrieval‐Augmented Generation** ^**[**^ ^27^ ^**]**^ **for Key Search Points (Algorithm** **S2** **, Supporting Information)**:
  (a) Doc Searchers: Within the established vector knowledge repository, we perform targeted searches at each key search point, selecting the top five (K = 5) outcomes as potential knowledge candidates.
  (b) Web Searcher: Concurrently, the LLM leverages its self‐retrieval capabilities to probe the web and online databases for crucial nodes. The data retrieved by the LLM are then aggregated and distilled into a concise knowledge synopsis for each node.
(5) **Knowledge Transfer Rules**: Drawing upon the knowledge acquired in the third stage, the LLM delineates precise filtering rules for potential experimental designs. These rules establish a framework for assessing the the viability of experimental candidate, guaranteeing both the clarity and practicality of the selection rules (See Algorithm S3, Supporting Information).
(6) **Candidates Evaluation**: Each experimental candidate derived from the preliminary screening of the reverse design process is individually scored. The candidate that attains the highest score and demonstrates the most logical rationale is chosen for subsequent experimental preparation and validation. The scoring rules encompass theoretical calculations of the candidate alloy, established material properties, and conformance with the anticipated performance model, among other factors.

To ensure the effectiveness of the screening process, we selected the currently most powerful and widely recognized LLMs, GPT‐4‐Turbo.^[^ ^22^ ^]^ The implementation of this process significantly improves the efficiency and precision in selecting experimental candidates, while also introducing an innovative automated tool for material design candidate selection. Moreover, it reduces the need for extensive expert involvement in the screening and evaluation of candidates, thereby diminishing the risks and costs associated with unreliable experimental candidates. Our proposed method significantly accelerates the process of screening experimental candidates, achieving a speed of ≈3 s per candidate in a single‐threaded process. This speed can be further increased with the addition of more processing threads.

### 2.2 Experimental Dataset

**Table** **1** provides a comprehensive overview of the HEAs dataset attributes, including the source domain S for HV and the target domains T for UTS and EL. The source domain dataset S only includes the composition features of the HEAs, with 23 dimensions in total, and a single target property, hardness value (HV). A dataset comprising 290 samples is present, and statistical information for each feature is available in Table S1 (Supporting Information). The target domain dataset T comprises a total of 90 samples that feature seven 7‐dimension composition features besides the 3‐dimension features of cold rolling, heat treatment temperature, and heat treatment time, on top of having two target properties, namely ultimate tensile strength (UTS) and elongation (EL). A total of 90 samples comprise the dataset, and statistical information for each feature is shown in Table S2 (Supporting Information).

**Table 1:** Comprehensive Overview of HEAs Dataset Attributes: Source Domain S and Target Domains T.

Attribute Name	Description	Unit	Mean	Std	Min	Max
S ***Hardness (HV) HEAs dataset***						
composition	Chemical composition (23 possible elements)	%	—	—	—	—
HV	Hardness	kgf mm^−2^	462.6	179.46	110	959.6
T ***UTS and EL HEAs dataset***						
composition	Chemical composition (7 possible elements)	%	—	—	—	—
cr	Cold Rolling	%	17.35	30.17	0	90
TAN	Heat processing temperature	°C	175.28	346.85	25	1200
tAN	Heat processing time	hours	0.19	0.49	0	2
UTS	Ultimate tensile strength	MPa	778.36	301.39	368	1800
EL	Elongation	%	25.29	19.51	0.2	78

We identified that datasets S and T do not align well because of differences in their composition features, where T has a subset of S’s feature space. Padding the datasets to align the features could result in sparse matrices in the target domain, which is unfavorable for building machine learning models with high precision. Therefore, we adopted the composition expansion method^[^ ^25^ ^]^ to densify the sparse feature matrices. We utilized atomic statistical features to generate high‐dimensional, information‐rich images. Moreover, to achieve feature alignment, we mapped the composition features of both S and T to grayscale images arranged in a regular 24 × 21 pattern. This feature alignment is an ideal foundation for manual construction of statistical information in transfer learning. Furthermore, the expansion method has several additional benefits beyond feature alignment, which we discussed in detail in Section S3.2 (Supporting Information).

### 2.3 Experimental Results of DSMID Framework

#### 2.3.1 The Overall Performance of AAEG

During the training process of the domain adaptation, we opted to use the binning task for the HV of HEAs to train the model, instead of directly performing regression tasks for HEAs performance prediction (for detailed reasons, please refer to Section S4.1, Supporting Information). The detailed training losses and bucketing accuracy of AAEG can be found in Figure S3 (Supporting Information).

The domain adaptation capabilities of our AAEG model are effectively demonstrated by the t‐SNE visualizations of component feature vectors from both the source and target domains, as shown in Figure S4 (Supporting Information). These visualizations highlight AAEG's success in reducing domain shift and enhancing transferability.

We further utilized the material composition embeddings output by AAEG for training regression tasks targeting three key performance metrics of HEAs (HV, UTS and EL). Both cross‐validation and out‐of‐distribution (OOD) data validated the effectiveness of our AAEG in representing material component embeddings.

**AAEG Embeddings for Material Property Prediction**. To validate the effectiveness of the material composition embeddings generated by AAEG, we input these embeddings into downstream regression models for testing. We selected three classic regression models: Random Forest (RF), eXtreme Gradient Boosting (XGBoost), and ensemble deep RVFL network (edRVFL, as described in Section S4.2.4, Supporting Information),^[^ ^28^ ^]^ representing tree‐based, ensemble, and vector‐based models, respectively.

The commonly used *R* ^2^ metric in regression tasks was employed as the evaluation criterion (for detailed definitions of the evaluation metrics, see Section S4.5, Supporting Information). Our method demonstrated exceptional performance in predicting the three objective performance criteria and thus confirmed its efficacy (refer to **Table** **2** for experimental results).

**Table 2:** *R* ^2^ Performance Comparison of Regression Models with Original Component Vectors and AAEG Representation Embeddings.

Input	Regression Model	HV	UTS	EL
Original component vectors	RF	0.71	0.228	0.457
	XGBoost	0.771	0.178	0.589
	edRVFL	0.789	0.566	0.691
AAEG representation embeddings	RF	0.873	0.575	0.7
	XGBoost	0.88	0.601	0.724
	edRVFL	0.919	0.804	0.849

**Figure 4:**
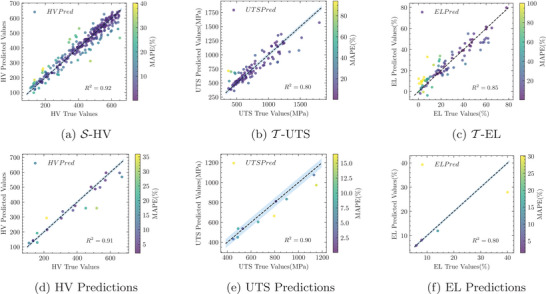
(Top row) Enhanced prediction accuracy in HEAs through AAEG: a tenfold validation analysis. (Bottom row) Comparative analysis of predictive and actual mechanical properties in OOD HEAs with the AAEG.

Based on the results of the original composition feature inputs, Table 2 lists the *R* ^2^ values of the best edRVFL model for the S‐HV, T‐UTS, and EL objective performance standards, which are 0.789, 0.566, and 0.691, respectively. There is a significant accuracy gap between the source domain and the target domain. To improve the predictive accuracy of the model in both domains, we adopted a domain adaptation method–AAEG. The AAEG representation embeddings significantly improved the *R* ^2^ values for all three objective standards, particularly for S‐HV and T‐UTS in the source and target domains, with improvements exceeding 10%. The transfer learning embeddings of AAEG effectively enhanced the prediction accuracy for different performance standards of the same material. Additionally, using the same model parameter settings, the RF and XGBoost models also showed significant improvements in regression prediction accuracy. **Figure** **4** presents the specific prediction scenarios for the three objective performance standards under tenfold cross‐validation, further verifying the effectiveness of the AAEG method. We compared AAEG with other transfer learning methods in Section 4.2.2, and the detailed comparison of results is presented in **Table** **3**.

**Table 3:** Results of the experimental *R* ^2^ on the HEA dataset. Shallow Transfer Learning and Deep Domain Adaptation lack experimental results on the original dataset since there is a need for two datasets for testing. Consequently, we obtained the ML outcomes for the transfer dataset using the AAEG network‐generated embedding. As for the edRVFL results, they are presented directly in the AAEG column.

	Method	HV	UTS	EL	HV (S→T)	UTS (S→T)	EL (S→T)
ML	RF	0.71	0.228	0.457	0.873	0.575	0.7
	XGBoost	0.771	0.178	0.589	0.88	0.601	0.724
	edRVFL	0.789	0.566	0.691	—	—	—
Shallow Transfer Learning	TCA	—			0.787	0.597	0.633
	TrAdaBoost				0.791	0.549	0.663
	CORAL				0.814	0.642	0.67
	BDA				0.814	0.637	0.775
Deep Domain Adaptation	Deep CORAL				0.811	0.626	0.808
	DANN				0.82	0.653	0.799
	CDAN				0.817	0.694	0.784
	AAEG				0.919	0.804	0.849

To demonstrate the generalization ability of our AAEG, we collected data from recent HEA literature, organizing 20 HV data points, 10 UTS data points, and 4 EL data points, all of which are OOD data (for detailed data and prediction results, see **Tables** **4**, **5**, and **6** in Section 4.2.3). The AAEG also performed excellently in predictive accuracy on these data, with a MAPE of 11.26%, MSE of 3053, and an *R* ^2^ of 0.91 for HV prediction (see Figure 4d). Additionally, the UTS prediction had a MAPE of 7.5%, MSE of 6328.20 (see Figure 4e), and an *R* ^2^ of 0.90, while the EL prediction had a MAPE of 12%, MSE of 37.52, and an *R* ^2^ of 0.80 (see Figure 4f). These findings emphasize the generalization and effectiveness of the AAEG in predicting mechanical properties related to the HEA system.

**Table 4:** Evaluating HV performance in HEAs from various sources using the AAEG, achieved results with *R* ^2^ = 0.91, *MSE* = 3053, and MAPE=11.26%. Here, AC: Arc Melting, SS: Solid Solution, IM: Intermetallic).

HEAs	Treatment	Phases	HV		Ref.
			True	Pred	
FeNiCrCoAl0.25	AC	SS	110	126	[29]
FeNiCrCoAl0.5	AC	SS	159	192	[29]
FeNiCrCoAl0.75	AC	SS	388	375	[29]
FeNiCrCoAl0.875	AC	SS	538	498	[29]
FeNiCrCoAl1	AC	SS	484	502	[29]
FeNiCrCoAl2	AC	SS	509	487	[29]
CoCrFeNi	AC	SS	160	129	[29]
CoCrFeMnNi	Annealed	SS	135	142	[30]
FeCrNiCoAl0.7Cu0.5	AC	SS + IM	313	337	[31]
FeCrNiCoAl0.75Cu0.25	AC	SS + IM	375	345	[31]
Al0FeMnNiCrCu0.5	AC	SS	216	293	[32]
Al0.25FeMnNiCrCu0.5	AC	SS	305	292	[32]
Al0.5FeMnNiCrCu0.5	AC	SS+ IM	452	359	[32]
Al0.5FeMnNiCrCu0.5	AC	SS + IM	518	359	[32]
Al37.5Cr12.5Cu12.5Fe12.5Ni25	AC	SS + IM	570	546	[33]
Al37.5Cr25Cu12.5Fe12.5Ni12.5	AC	SS + IM	574	596	[33]
Al37.5Cr12.5Cu12.5Fe25Ni12.5	AC	SS + IM	656	596	[33]
Al37.5Cr12.5Cu25Fe12.5Ni12.5	AC	SS + IM	670	568	[33]
FeCoCrNiMn	Annealed	SS	135	142	[34]
Al0.5CoCrFeCuNi	Annealed	SS	219	214	[35]

**Table 5:** Assessing UTS performance of HEAs through the application of the AAEG in other studies, achieved Results with *R* ^2^ = 0.90, *MSE* = 6328, and MAPE=7.5%.

HEAs	Phases	Treatment				UTS		
		Route	cr	TAN	tAN	True	Pred	Ref.
CoCrFeMn	—	AC	0	25	0	795	663	[36]
Co9Cr9Fe9Mn9Ni4	—	AC	0	25	0	450	431	[36]
CoCrFeMnNi	—	AC	0	25	0	530	537	[36]
CoCrFeAlNi1.5	—	AC	0	25	0	1150	973	[36]
CoCrFeAlNi2	—	AC	0	25	0	1130	1075	[36]
CoCrFeAlNi2.5	—	AC	0	25	0	900	832	[36]
CoCrFeAlNi3	—	AC	0	25	0	810	810	[36]
CoCrFeAlNi4	—	AC	0	25	0	660	603	[36]
CoCrFeNi	SS	AC	0	25	0	488	449	[34]
CoCrFeNiMn	SS	AC	0	25	0	491	537	[34]

**Table 6:** EL performance prediction for HEAs by utilizing the AAEG in literature analysis, achieved results with *R* ^2^ = 0.80, *MSE* = 37, and MAPE=12%.

HEAs	Phases	Treatment				EL		
		Route	cr	TAN	tAN	True	Pred	Ref
CoFeNi2Al0.9	SS	AC	0	25	0	40	27	[37]
CrFeNi2Al	SS	AC	0	25	0	6	5	[38]
AlCoCrFeNi2.1	SS	Annealed	90	800	1	14	11	[39]
AlCoCrFeNi2.1	SS	Annealed	90	600	1	8	8	[40]

#### 2.3.2 The Overall Performance of AMSEP

**Designing and Screening HEAs through AMSEP**. Discovering high‐performing HEAs is an arduous task due to their complex composition and structural diversity. Nevertheless, through the aforementioned experiments, we have acquired an edRVFL model capable of accurately predicting the UTS and EL of HEAs. Recently, EHEAs have attracted more and more attention due to their excellent mechanical properties. The optimal as‐cast EHEAs exhibit UTS and EL approximating 1*GP* and 18%, respectively, in the dataset. Our objective is to reverse‐engineer and develop a novel system through screening evaluations with a composition capable of attaining the highest performance within the dataset. We employ the model as a discriminator by implementing the NSGA‐II algorithm^[^ ^21^ ^]^ (Detailed parameters for NSGA can be found in Table S8, Supporting Information.), and leverage the GPT‐Base pipeline to screen and assess candidate experimental candidates. This methodology facilitates the design of potential EHEA alloys with properties that meet expectations (*UTS* ∼ 1000*MPa*, EL∼20%).

**Figure 5:**
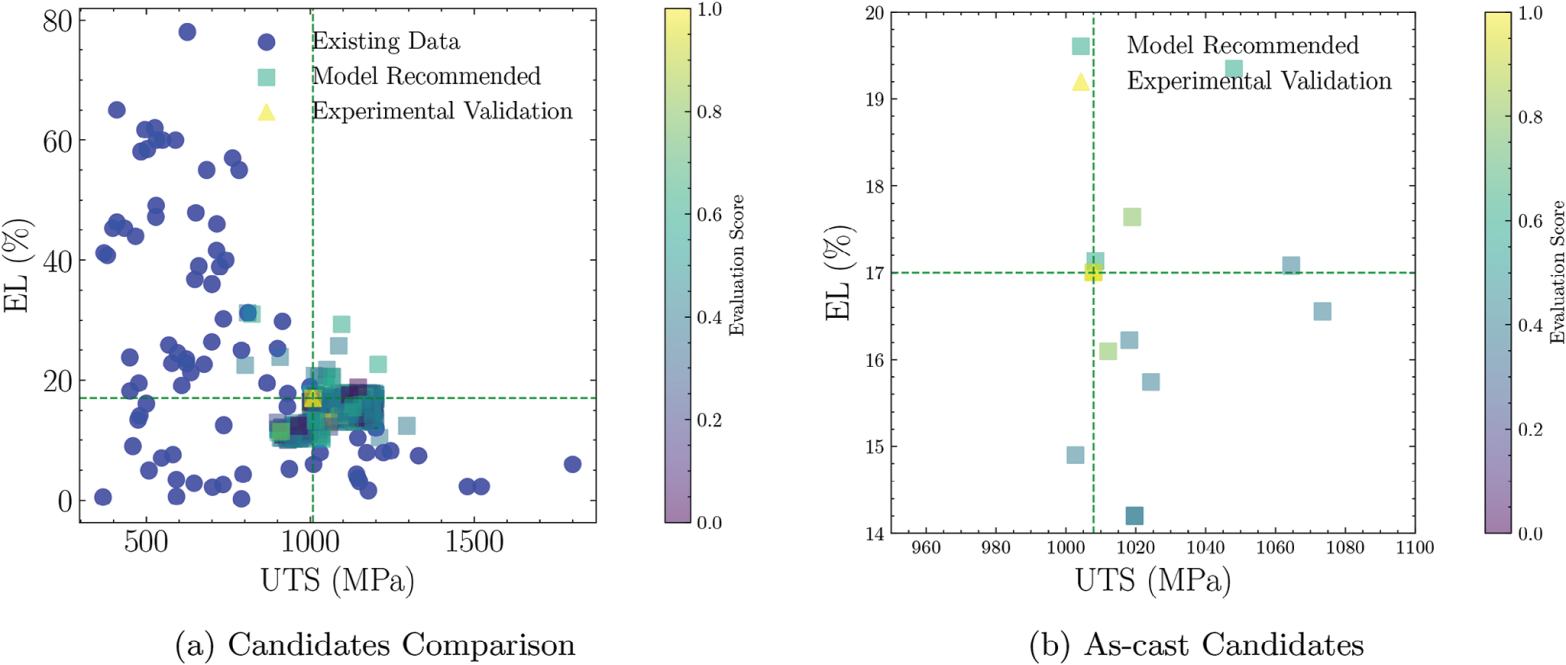
Overview of the Inverse Design Results Evaluated Using AMSEP. a) Comparison of experimental candidates with existing data points in the dataset. b) Evaluation of experimental schemes without additional processing. The mean UTS of this design outcome is 1071.36 MPa, while the mean EL is 16.36%.

Table S9 (Supporting Information) displays the ranges for each feature in the gene pool of the NSGA‐II algorithm. This algorithm is applied in these gene pools to generate several solutions, each representing the possible combinations of HEAs compositions and heat treatment. The UTS and EL performance objectives of these solutions are evaluated using the edRVFL model. Subsequently, these solutions are sorted based on their non‐dominant level and crowding distance. Following N iterations of the algorithm, we produced 1030 experimental candidates via a reverse design that aligned with our criteria. Given the volume, manual selection of the most valuable candidates among them is virtually untenable. Consequently, we formulated six key search points grounded in expert knowledge and our objectives. GPT‐4‐Turbo^[^ ^22^ ^]^ initially employed these points to filter the 1030 candidates based on pertinent knowledge and the latest experimental findings, culminating in a shortlist of 362 viable candidates. Concurrently, GPT‐4‐Turbo applied a relevance filter to the 169 papers we submitted, from which 46 pertinent studies were segmented and vectorized to establish a related vector knowledge base. GPT‐4‐Turbo then integrated information from Web and Document Searches to devise evaluation criteria for the candidates, assigning scores to each of the 362 candidates and elucidating the rationale behind each evaluation (**Figure** **5**).

From Figure 5a, it is evident that our recommended candidates are positioned in the upper right quadrant of the original data, indicating superior comprehensive mechanical properties relative to other entries. Figure 5b reveals that our selected candidate for experimental validation, without additional processing, scores the highest and exhibits exceptional performance.

**Experimental Synthesis and Validation** Ultimately, from the three top‐rated experimental designs, we chose the one that avoids heat treatment and additional preparatory processes (Al_14_(CoCrFe)_19_Ni_28_). This choice was driven by its ability to attain performance comparable to alternative designs without complex procedures and its alignment with green manufacturing principles. Moreover, this experiment requires simpler preparation and validation. The most famous EHEA system is the AlCoCrFeNi series HEAs including AlCoCrFeNi_2.0_, AlCoCrFeNi2.1, and AlCoCrFeNi_2.2_.^[^ ^24^ ^]^ Therefore, the Al_14_(CoCrFe)_19_Ni_28_ is a potential EHEA, and the mechanical properties might be good as our predicting. Detailed evaluation scores and rationales are available in Table S10 (Supporting Information).

We synthesized Al14(CoCrFe)19Ni_28_ and measured its corresponding properties. The AAEG predictions for this EHEA show a UTS of 1008 MPa and EL of 17%, closely approximating the experimentally measured values of 1085 MPa and 23.8%. This validates the model's accuracy in simulating real‐world alloy behaviors without the need for complex synthesis processes.

**Figure 6:**
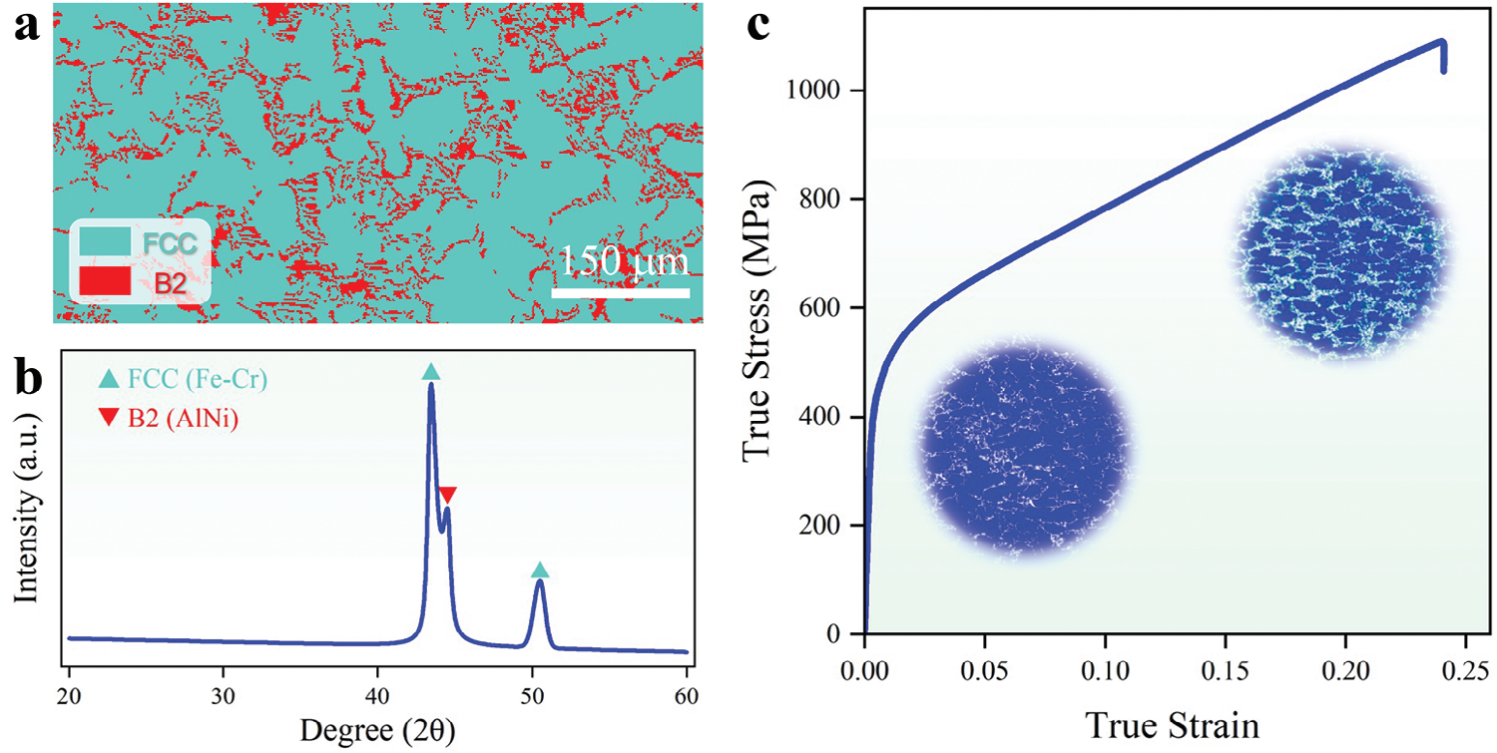
The microstructure and true stress versus true strain curves of as‐cast design alloy: a) Electron backscatter diffraction (EBSD) phase image, b) X‐ray diffractometer (XRD) pattern. c) True stress versus true strain curves of the design HEA with a strain rate of 10^−3^ *s* ^−1^, the inset is the geometrically necessary dislocations (GND) density of design HEA before and after tension. Detailed microstructural changes in Al_14_(CoCrFe)_19_Ni_28_ EHEA under tension can be found in Section S7 (Supporting Information).

**Figure** **6**a displays the Electron backscatter diffraction phase image of the newly‐design Al_14_(CoCrFe)_19_Ni_28_ HEA, a typical eutectic microstructure can be observed via microscope, the coarse FCC phases are surrounded by lamella B2 phases. Then, the XRD pattern in Figure 6b further indicated a mixture of FCC and B2 (ordered BCC) crystal structure for this alloy (The detailed analysis methods for this alloy are described in Section 4.3).

Figure 6c presents the tensile behavior of our newly‐design EHEA, Al_14_(CoCrFe)_19_Ni_28_, at ambient temperature, indicating a high strength and good ductility. The yield stress, UTS, and EL of newly‐design EHEA are 362 MPa, 1085 MPa, and 23.8%, respectively. The soft FCC grains deform easily and thus bear more plastic strains than hard BCC grains. The huge amounts (86%) of soft FCC phases lead to the large fracture elongation. The plastic strain gradients exhibit in the soft FCC matrix near the FCC/B2 interface, and the accommodation of the strain gradients requires the storage of geometrically necessary dislocations (GND),^[^ ^41^ ^]^ leading to the back‐stress hardening. See the inset of Figure 6c, the GND density near the FCC/B2 interface is much greater than that in the inner FCC grains. Similarly, the storage of GND also can be noticed in B2 phase near the interface (See Figure S6, Supporting Information). Therefore, an ultra‐high strain hardening phenomenon can be observed in the stress–strain curve.

The newly proposed EHEA Al_14_(CoCrFe)_19_Ni_28_ demonstrates significantly greater plasticity without strength loss compared to the typical as‐cast EHEA AlCoCrFeNi_2.1_.^[^ ^24^ ^]^ Alloys with high plasticity exhibit remarkable ductility and toughness, allowing for substantial deformation without fracturing, which enhances their suitability for critical applications across various applications, including automotive, aerospace, marine, and petroleum industries. Their superior formability and resistance to cracking not only extend the lifespan of components but also reduce manufacturing costs, positioning them as valuable materials in the advancement of industrial technologies.

## 3 Conclusion

In our study, we introduced an innovative Adversarial domain Adaptive Embedding Generative network (AAEG) that merges adversarial learning with representation learning, effectively enhancing domain adaptability for small datasets. By integrating residual connections and Deep CORAL Loss into a generator‐discriminator framework, we facilitated improved gradient updates and minimized domain shift, leading to enhanced performance in regression models. This approach not only addresses the convergence challenges of GAN‐based domain adaptive frameworks but also shifts the focus toward more effective material composition representation learning. The universality of the composition in the material datasets highlights the robust generalization of AAEG within the materials science domain.

The remarkable material characterization data from AAEG have yielded a multitude of reverse design results. To tackle the challenge of identifying the most promising reverse design candidates, we proposed an LLM‐based Automated Material Screening and Evaluation Pipeline (AMSEP) for experimental candidates. The implementation of this automated selection and evaluation process is a major advancement in expediting material research and innovation. Integrating multi‐source data processing, intelligent literature review, critical information extraction, and data‐driven decision‐making, this approach significantly enhances the efficiency of experimental candidates identification and diminishes research expenditures.

We have integrated AAEG and AMSEP into a cohesive framework referred to as the Dual‐Strategy Materials Intelligent Design (DSMID) Framework. AAEG provides AMSEP with reliable performance predictions, enabling rapid screening and evaluation of promising experimental candidates. This synergy facilitates a closed‐loop system for intelligent material design. Employing the DSMID Framework, we devised and predicted a novel EHEA material exhibiting a UTS of ≈1.1*GPa* and an EL of ≈24% which is exceptional among the reported as‐cast EHEAs. This enhances their suitability for critical applications across various applications, including automotive, aerospace, marine, and petroleum industries.

Despite the promising results, our study has several limitations that need to be addressed in future research. One limitation is that the current transfer learning is applied between two different properties of the same material, and its effectiveness across different materials still requires further validation. Additionally, the performance of general large language models in specific domains is limited by their broad knowledge base. Future work could focus on developing specialized domain‐specific models to increase knowledge depth and improve screening effectiveness.

The future of materials science offers numerous opportunities for methodological advancements. A promising avenue involves integrating sophisticated encoder architectures and refining our approaches to better navigate the intricate challenges of domain adaptability. Specifically, domain adaptation techniques can be applied to uncover latent correlations between materials datasets across varied contexts.

Concurrently, the development of a language model‐based screening and evaluation pipeline for experimental candidates, augmented by a user feedback system, represents a valuable research trajectory. Crafting domain‐specific language models could substantially enhance their comprehension and interpretive capacities, thereby streamlining and expediting reverse engineering.

## 4 Experimental Section

#### Training Process for AAEG Model

A detailed explanation of the training process for the four networks is provided. To achieve combined learning of component representation embedding generation and GAN, the training process for the *D*, *G*, *F*, and *C* networks is set:

First, the input of the discriminator *D*, combined with the residual connection of the generator *F*, is divisible into three segments: 1) the genuine image input x necessitates correct scrutiny from *D* relative to its provenance (if it arises from the source field, it ought to be authentic, otherwise treated as counterfeit from the target field) and requires the synchronous production of *D*_*cls*_(*x*) Equation (1); 2) *D* needs to confirm the falsity status of the image, *G*(*x*_*g*_), generated by *G*; therefore, the gradients are not required to backpropagate in *D* while computing *D*_*cls*_(*G*(*x*_*g*_)) Equation (2); 3) the third segment entails the output of *F*, *F*(*x*), and requires *D* to accurately recognize its source in terms of the field, while producing the corresponding *D*_*cls*_(*x*) Equation (3). It is important to note that gradient backpropagation herein entails only the fully connected layer of D's network, and the gradients do not backpropagate to the CNNs layer.

(1) $$L_{D}^{x}=f_{L}\left(D_{cls}\left(x\right),\;L\right)+f_{B}\left(D_{data}\left(x_{S}\right),\;Y_{T}\right)\;+\;f_{B}\left(D_{data}\left(x_{T}\right),\;Y_{F}\right)$$

(2) $$L_{D}^{G(x_{g})}=f_{B}\left(D_{data}\left(G(x_{g})\right),Y_{F}\right)$$

(3) $$\begin{array}{ccc} L_{D}^{F\left(x\right)} & = & f_{L}\left(D_{cls}\left(F\left(x\right)\right),\;L\right)+f_{B}\left(D_{data}\left(F(x_{S})\right),\;Y_{T}\right)+\\ & & f_{B}\left(D_{data}\left(F(x_{T})\right),\;Y_{F}\right) \end{array}$$

where *f*_*L*_ represents the cross‐entropy loss, whereas *f*_*B*_ represents the binary cross‐entropy loss.

Second, in order to produce more realistic images, *G* utilizes the gradient of *D* to perform updates using a combination of adversarial loss and classification loss Equation (4).

(4) $$L_{G}=f_{B}\left(D_{data}\left({G(x}_{g})\right),Y_{T}\right)+f_{L}\left(D_{cls}\left({G(x}_{g})\right),\;L\right)$$

Third, network *C* exclusively accepts the source domain embedding $F(x_{S})$ derived by F, and updates via traditional supervised learning using source images and labels Equation (5).

(5) $$L_{C}=f_{L}\left(C\left(F\left(x_{S}\right)\right),\;L\right)$$

Finally, network *F*, as the most significant characteristic, directly influences the precision of regression prediction in network characterization. F receives gradients from sources *C*, *D*, and *G*, and undergoes CORAL Loss to minimize domain bias in the feature representation embedding. Specifically, *F* receives 1) the source‐domain bucketing loss from *C*, which suits the large data amount in the source domain for *F* to model the marginal distribution better and encourage the generalization ability of feature extraction through the gradients from *C* Equation (6); 2) gradients from *G*, which are crucial to providing *F* with semantic information to ensure the target embedding's consistency with the source distribution, thereby reducing domain shift Equation (7); 3) a residual connection between *F* and *D* is added since *G*'s training is oscillating on a small material dataset, *F* may be instable when receiving gradients from *D* through *G*, GRL is used to invert the losses gradient in D, and eventually transmitted to *F* Equation (8); 4) CORAL Loss can reduce the discrepancy between two domain embeddings. However, during the initial stage, convergence on *C* and *D* is crucial for adequately bucketing the feature embeddings. This need arises as CORAL Loss must be dynamically weighted to balance domain shift and label allocation Equation (9). The mathematical representation of the four loss components is shown below:

(6) $$L_{F}^{C}=f_{L}\left(C\left(F\left(x_{S}\right)\right),\;L\right)$$

(7) $$L_{F}^{G}=\alpha *\left(f_{B}\left(D_{data}\left({G(x}_{g})\right),Y_{T}\right)+f_{L}\left(D_{cls}\left({G(x}_{g})\right),\;L\right)\right)$$

(8) $$\begin{array}{ccc} L_{F}^{D} & = & {\beta *(f}_{L}\left(D_{cls}\left(F\left(x\right)\right),\;L\right)+f_{B}\left(D_{data}\left({F(x}_{S})\right),\;Y_{T}\right)+\\ & & f_{B}\left(D_{data}\left({F(x}_{T})\right),\;Y_{F}\right)) \end{array}$$

(9) $$L_{CORAL}=\lambda *\frac{1}{4d^{2}}{\left\|C_{x_{S}}-C_{x_{T}}\right\|}_{F}^{2}$$

where α, β, and λ represent the weight coefficients of the adversarial loss for target, the residual connection loss, and the CORAL loss, respectively. Notably, λ is proportional to the number of training rounds. When back‐propagated to *F*, the gradient of the loss function $f_{B}\left(D_{data}\left({F(x}_{T})\right),\;Y_{F}\right)$, is reversed via Gradient Reversal Layer (GRL). Consequently, the goal of loss optimization is to align the feature distributions of the source and target domains.

Algorithm 1 **Iterative training procedure of our approach.**

**Table:** 

1:	**Let** training iterations = N
2:	**while** epoch<=N **do**
3:	Sample *k* images with labels from target domain $T:{\left\{t_{i},y_{i}\right\}}_{i=1}^{k}$
4:	Let *h*_*i*_ = *F*(*t*_*i*_) be the embeddings computed for the target images.
5:	Sample *k* images with labels from source domain $S:{\left\{s_{i},y_{i}\right\}}_{i=1}^{k}$
6:	Let *f*_*i*_ = *F*(*s*_*i*_) be the embeddings computed for the source images.
7:	Sample *k* random noise samples ${\left\{z_{i}\right\}}_{i=1}^{k}∼N\left(0,1\right)$
8:	Let $f_{g_{i}}$ and $h_{g_{i}}$ be the concatenated inputs to the generator.
9:	Let *x* = {*s*, *t*}, *v* = {*h*, *f*}, *x*_*g*_ = {*f*_*g*_, *h*_*g*_}
10:	Update discriminator using the following objectives: (10) $L_{D}=L_{D}^{G(x_{g})}+L_{D}^{x}+L_{D}^{F(x)}$ (11) $$\begin{array}{c} L_{D}^{G(x_{g})}=\max_{D}\log\left(1-D_{\text{data}}\left(x_{g}\right)\right)\\ L_{D}^{x}=\max_{D}\frac{1}{2k}\sum_{i=1}^{2k}\log\left(D_{cls}\left(x_{i}\right)\right)+\frac{1}{k}\sum_{i=1}^{k}\log\left(D_{\text{data}\;}\left(s_{i}\right)\right)\\ +\log\left(1-D_{\text{data}\;}\left(t_{i}\right)\right)\\ L_{D}^{F(x)}=\max_{D}\frac{1}{2k}\sum_{i=1}^{2k}\log\left(D_{cls}\left(v_{i}\right)\right)+\frac{1}{k}\sum_{i=1}^{k}\log\left(D_{\text{data}\;}\left(f_{i}\right)\right)\\ +\log\left(1-D_{\text{data}\;}\left(h_{i}\right)\right) \end{array}$$
11:	Update the generator, through the discriminator gradients computed using real labels: (12) $L_{G}=\min_{G}\frac{1}{2k}\sum_{i=1}^{2k}-\log\left(D_{cls}\left(G\left(v_{i}\right)\right)\right)+\log\left(1-D_{\text{data}\;}\left(G\left(v_{i}\right)\right)\right)$
12:	Update the classifier C for the source data using a cross‐entropy loss function: (13) $L_{C}=\min_{C}\frac{1}{k}\sum_{i=1}^{k}-\log\left(C\left(f_{i}\right)\right)$
13:	Update the embedding F using a linear combination of the adversarial loss, classification loss, and CORAL loss (when input *t*_*i*_, set *GRL* = *True*): (14) $L_{F}=L_{F}^{C}+\alpha *L_{F}^{G}+\beta *L_{F}^{D}+\lambda *L_{CORAL}$ (15) $$\begin{array}{c} L_{F}^{C}=\min_{F}\frac{1}{k}\sum_{i=1}^{k}-\log\left(C\left(f_{i}\right)\right)\\ L_{F}^{G}=\min_{F}\frac{1}{2k}\sum_{i=1}^{2k}-\log\left(D_{cls}\left(G\left(v_{i}\right)\right)\right)+\log\left(1-D_{\text{data}\;}\left(G\left(v_{i}\right)\right)\right)\\ L_{F}^{D}=\min_{F}\frac{1}{2k}\sum_{i=1}^{2k}-\log\left(D_{cls}\left(v_{i}\right)\right)+\max_{F}\frac{1}{k}\sum_{i=1}^{k}\log\left(D_{\text{data}\;}\left(f_{i}\right)\right)\\ \;+\log\left(1-D_{\text{data}\;}\left(h_{i}\right)\right)\\ L_{CORAL}=\frac{1}{4d^{2}}{\left\|C_{f}-C_{h}\right\|}_{F}^{2} \end{array}$$
14:	**end while**

#### Comparative Analysis and Experimental Results of AAEG

To comprehensively evaluate the predictive performance of our AAEG model for material property predictions, the experiments were designed into two primary categories: Cross‐Validation and Out‐of‐Distribution (OOD).

The Cross‐Validation Performance experiments aim to validate the model's efficacy within the known data distribution, providing insights into its reliability and robustness during training. This process ensures that the model performs well on data it has encountered during training.

Conversely, the OOD Performance experiments assess the model's ability to generalize to unseen data, which is critical for evaluating its real‐world applicability and potential for discovering novel materials with unique properties. By addressing both known and unknown data distributions, this dual evaluation framework offers a comprehensive and rigorous analysis of the model's capabilities.

The impact of each component of the AAEG model on predictive accuracy was examined through ablation experiments. Detailed results of these experiments are provided in Table S6 (Supporting Information).

##### Baseline

We compared the experimental results of AAEG with those of typical machine learning, non‐deep transfer learning, and deep domain adaptive algorithms. These baselines were selected to provide a comprehensive evaluation across different levels of model complexity and adaptability to domain shifts.

1) Traditional machine learning: RF,^[^ ^42^ ^]^ XGBoost,^[^ ^43^ ^]^ edRVFL.^[^ ^28^ ^]^
2) Shallow transfer learning: Transfer component anaysis (TCA),^[^ ^44^ ^]^ TrAdaBoost,^[^ ^45^ ^]^ CORrelation alignment(CORAL),^[^ ^46^ ^]^ Balanced distributon adaptation (BDA).^[^ ^47^ ^]^
3) Deep domain adaptation: Deep CORAL,^[^ ^48^ ^]^ DANN,^[^ ^9^ ^]^ Conditional adversarial domain adaptation (CDAN).^[^ ^10^ ^]^

We trained a total of seven migration learning models to compare against AAEG. This includes four shallow transfer learning models and three deep domain adaptation models. Each of these models represents different strategies and methodologies for handling domain shifts and enhancing model generalization:

1) Shallow transfer learning models focus on feature transformation and re‐weighting strategies to align the source and target distributions.
  (a) Transfer Component Analysis^[^ ^44^ ^]^ transforms data from both domains into a common subspace.
  (b) TrAdaBoost^[^ ^45^ ^]^ re‐weights instances from the source domain to better match the target domain.
  (c) CORAL^[^ ^46^ ^]^ aligns the second‐order statistics of the source and target domains.
  (d) Balanced Distribution Adaptation^[^ ^47^ ^]^ balances the marginal and conditional distributions between domains.
2) Deep domain adaptation models leverage deep learning techniques to learn domain‐invariant representations.
  (a) Deep CORAL^[^ ^48^ ^]^ extends CORAL by incorporating it into a deep neural network.
  (b) Domain‐Adversarial Neural Network^[^ ^9^ ^]^ uses adversarial training to learn domain‐invariant features.
  (c) Conditional Adversarial Domain Adaptation^[^ ^10^ ^]^ conditions the domain discriminator on both feature representations and classifier predictions.

The code was sourced for the shallow transfer learning and deep domain adaptation methods.^[^ ^49^ ^]^ Notably, the original code for shallow transfer learning methods, which was primarily designed for classification tasks and heavily relied on KNN and tree models, was replaced with the edRVFL regression model to enable better comparability in materials property regression tasks. The same model was also used in the AAEG algorithm.

The previous experiment for the deep domain adaptation method also focused on classification tasks, but to ensure fair comparison with AAEG, we trained the model using bucket labels for performance and edRVFL regression models for materials property prediction based on the domain‐adaptive embeddings. To more accurately evaluate the performance and generalization ability of our models, we calculated evaluation metrics by averaging over ten‐fold cross‐validation runs conducted on the dataset.

##### Cross‐Validation of AAEG

First, the accuracy of three ML models on the non‐transferred dataset was used for the baseline (The detailed description of the baseline is provided in Section 4.2.1). Subsequently, we trained eight transfer learning models to obtain transferred embeddings, which were then used as inputs to the edRVFL for predicting regression tasks and corresponding *R* ^2^ values. Our method demonstrated exceptional performance in predicting the three objective performance criteria and thus confirmed its efficacy (refer to Table 3 for experimental results).

*Shallow transfer learning* and *Deep domain adaptation* methods learned the domain shift between S and T to obtain better compositional representation feature embeddings. Consequently, there was a varied improvement in the *R* ^2^ values. The AAEG model outperformed the other models, achieving the highest *R* ^2^ values for HV, UTS, and EL. These results demonstrate the effectiveness of the AAEG model in generating embeddings that enhance the performance of the edRVFL model in predicting material properties.

##### External Validation of AAEG

External validation of the AAEG framework is conducted using the latest experimental data from recent HEA research publications. This approach ensures the model's efficacy in predicting HV, UTS, and EL properties of HEAs across diverse sources. The results are presented in Tables 4, 5, and 6.

The results indicate that the AAEG demonstrates excellent accuracy in predicting all three performance metrics, particularly for HEAs with different processing methods and phases. The framework accurately predicts the actual performance values. Despite the limited data in the EL prediction experiments, the AAEG still showed good predictive performance, validating its effectiveness in small sample data scenarios. Overall, the AAEG exhibits outstanding predictive capabilities on in‐distribution data and maintains high reliability and accuracy on OOD small sample data. These results strongly support the wide application of the AAEG in practical scenarios.

#### Alloy Ingot Preparation and Analysis

Alloy ingots with a nominal composition of Al_14_(CoCrFe)_19_Ni_28_ were prepared by arc‐melting in a high‐purity argon atmosphere, where the elements with purities greater than 99.9 wt.% were employed as raw materials. The molten alloy was cast into a copper mold, and the ingots were remelted seven times to achieve chemical homogeneity. The 12 × 4 × 1.5 mm bar‐shaped specimens were cut from the as‐cast ingot via an electric discharging machine for the tensile tests. The tensile tests were performed under a strain rate of 10^−3^ *s* ^−1^ and repeated three times to ensure repeatability. The microstructural observations were conducted on an optical microscope and the electron backscatter diffraction (EBSD, EDAX Hikari XP). The crystal structure was identified using an X‐ray diffractometer (XRD, Bruker D8).

## Conflict of Interest

The authors declare no conflict of interest.

## Author Contributions

Y.Y. contributed to the writing of the original draft, data curation, software implementation, investigation, formal analysis, and visualization. J.X. was involved in data curation, investigation, experimental validation and analysis of high‐entropy alloys (HEA), as well as writing the review and editing. X.W. participated in the investigation and the review and editing of the writing. Q.Q. played a key role in conceptualization, methodology, funding acquisition, project administration, supervision, and also contributed to the review and editing of the writing.

## Supporting information

Supporting Information

## Data Availability

The data and source code that support the findings are available from the corresponding author upon reasonable request.
