# Supplementary material for: From Small Data Modeling to Large Language Model Screening: A Dual‐Strategy Framework for Materials Intelligent Design
Source: Adv Sci (Weinh). 2024 Oct 4;11(45):2403548. doi: 10.1002/advs.202403548 (PMC11615768; doi:10.1002/advs.202403548)
Supplement: Supplementary file 1 — Supporting Information [file ADVS-11-2403548-s001.pdf]

ADVANCED SCIENCE
Open Access

Supporting Information

for *Adv. Sci.*, DOI 10.1002/advs.202403548

From Small Data Modeling to Large Language Model Screening: A Dual-Strategy Framework for Materials Intelligent Design

Yeyong Yu, Jie Xiong, Xing Wu and Quan Qian**

From Small Data Modeling to Large Language Model Screening: A Dual-Strategy for Materials Intelligent Design

SUPPLEMENTARY INFORMATION

Yeyong Yu[1], Jie Xiong[2*], Xing Wu[1,3,4] and Quan Qian[1,2,3,4*]

[1]School of Computer Engineering & Science, Shanghai University, Shanghai, 200444, China.
[2]Center of Materials Informatics and Data Science, Materials Genome Institute, Shanghai University, Shanghai, 200444, China.
[3]Key Laboratory of Silicate Cultural Relics Conservation (Shanghai University), Ministry of Education, China.
[4]Shanghai Institute for Advanced Communication and Data Science, Shanghai University, Shanghai, 200444, China.

*Corresponding author(s). E-mail(s): xiongjie@shu.edu.cn; qqian@shu.edu.cn;
Contributing authors: yuyeyong@shu.edu.cn; xingwu@shu.edu.cn;

1 Related Work 2

2 Dataset Statistics and Notations 3

3 Preliminary Work for AAEG Training 3
- 3.1 HV mapping and bucketing from UTS . . . 3
- 3.2 Alignment component feature . . . 4

4 Details of AAEG 6
- 4.1 Domain Adaptation Training Task . . . 6
- 4.2 Auxiliary Methods Used in the AAEG Framework . . . 7
  - 4.2.1 Generative Adversarial Network . . . 7
  - 4.2.2 Deep CORAL Loss . . . 8
  - 4.2.3 Gradient Reversal Layer . . . 9
  - 4.2.4 Ensemble deep RVFL . . . 9
- 4.3 Network architecture details . . . 10
- 4.4 Training and Testing Process for AAEG . . . 11
- 4.5 Evaluation Metrics and Loss Function Setup . . . 11
- 4.6 Training Losses of AAEG . . . 11
- 4.7 Visualization of Domain Adaptation with AAEG. . . . 12
- 4.8 Ablation Study . . . 13
- 4.9 Correlation analysis between generated images and target performance . . . 14

5 Hyperparameter configuration **16**
5.1 AAEG parameters setting 16
5.2 NSGA-II parameters setting 17

6 Details of AMSEP **17**
6.1 Key Search Points and Evaluation Rules 17
6.2 Experimental Candidate Scheme Evaluation Results 18

7 Fabrication Methods and Microstructural Changes of $Al_{14}(CoCrFe)_{19}Ni_{29}$ EHEA Under Tension **20**

1 Related Work

Machine Learning Applied to HEAs and Involved in Reverse Design. Wen et al. proposed a machine learning-based strategy for designing high-entropy alloys(HEAs), which utilizes support vector machine and genetic algorithm to screen out HEAs with targeted properties, such as hardness, strength, and elongation, from a large number of candidates [1]. Houlong Zhuang provided a comprehensive overview of the latest research progress on HEAs in terms of concepts, classifications, synthesis, characterization, structure, and properties, as well as a prospective discussion on the integration of machine learning [2]. Feng et al. proposed a combined computational screening and experimental validation approach to rapidly design light-weight and low-cost HEAs with excellent strength and elongation [3]. Yang et al. constructed a hardness database, and established hardness prediction models using support vector machine (SVM) and artificial neural network (ANN), and then applied genetic algorithm (GA) for optimization search, discovering novel HEAs with ultra-high hardness [4].

Application of Transfer Learning in Materials Science. Due to the tremendous potential of transfer learning frameworks in overcoming the limited data availability problem in materials, there is an increasing application space for these frameworks in the high-cost experimental material field. Yoshida et al. developed a pre-trained model library using the OQMD and Material Project databases. The library contains over 140,000 pre-trained models for various properties of small molecules, polymers, and inorganic crystals. This reveals potential connections between small molecules and polymers, as well as between organic and inorganic chemistry [5]. Stephen Wu et al. established a neural network model between polymer structure and specific heat capacity, glass transition temperature, and melting point. They constructed a feature set and transferred it to modeling of polymer thermal conductivity, achieving the development of highly thermally conductive polymers [6]. Jha et al. used data from databases such as OQMD to build a calculation data model for the relationship between inorganic material composition and formation energy through machine learning. They then transferred the model parameters to experimental data-based modeling, significantly improving predictive ability in the formation energy model [7]. Xiang Li et al. trained a transfer learning machine learning model using physical knowledge structures and elemental descriptors to screen stable perovskite structures [8].

Domain Adaptation Methods. Domain Adaptation (DA) is a critical branch of feature transfer, and reducing the distribution changes between the $\mathcal{S}$ and $\mathcal{T}$ is the most effective and direct direction in domain adaptation methods. Transfer Component Analysis (TCA) is a feature-based data marginal distribution adaptation method that uses Maximum Mean Discrepancy (MMD) to obtain $\mathcal{S}$ and $\mathcal{T}$ feature representations with close distributions [9]. Deep Domain Confusion (DDC) jointly minimizes the classification task loss of the last fully connected layer and MMD loss [10]. Deep Adaptation Networks (DAN) extends this idea by embedding all task-specific layers into the kernel Hilbert space and minimizing MMD in the projection space [11]. Domain Adversarial Network (DANN) utilizes gradient reversal layers to align the distribution of features extracted by the model in the $\mathcal{S}$

and $\mathcal{T}$ and applies adversarial losses in the embedding space [12]. Sankaranarayanan et al. use AC-GAN to apply adversarial losses in the pixel space for domain adaptation [13]. Long et al. proposed a novel deep domain adaptation method, CDAN, that combines adversarial networks with multilinear conditioning for transferable representation learning [14]. Prototypical Contrast Adaptation (ProCA) incorporates inter-class information into the prototypes of classes and adapts using class-centered distribution alignment and contrastive learning [15]. Self-corrected unsupervised domain adaptation (SCUDA) proposes a self-correcting learning framework for end-to-end learning of target prediction without $\mathcal{S}$ classification and distribution alignment [16].

We propose a domain-adaptive method for predicting the performance of HEAs, which can generate excellent compositional embeddings of HEAs from very few data and show superior performance in subsequent property regression tasks. Based on high-precision performance models, we use a genetic algorithm to reverse design HEAs with high tensile strength and elongation, extrapolate the Pareto front of the dataset, and experimentally verify the results.

2 Dataset Statistics and Notations

Table S1 shows the statistics of $\mathcal{S}$ datasets and Table S2 shows the statistics of target datasets. Table S3 lists the notations used in this paper with descriptions. Table S4 provides a comprehensive list of abbreviations and their corresponding full terms used in the main text.

Table S1: Statistical information on the characteristics of the Hardness(HV) HEAs dataset $\mathcal{S}$.

Element	Description	mean	std	min	max
Al	Aluminum content	0.110	0.106	0	0.429
Co	Cobalt content	0.121	0.101	0	0.429
Cr	Chromium content	0.132	0.095	0	0.375
Fe	Iron content	0.147	0.096	0	0.476
Ni	Nickel content	0.150	0.103	0	0.385
Cu	Copper content	0.072	0.087	0	0.333
Mn	Manganese content	0.023	0.062	0	0.238
Si	Silicon content	0.007	0.030	0	0.200
V	Vanadium content	0.037	0.079	0	0.333
Mo	Molybdenum content	0.041	0.075	0	0.333
Ta	Tantalum content	0.021	0.060	0	0.250
Sn	Tin content	0.000	0.003	0	0.038
Ti	Titanium content	0.054	0.094	0	0.375
Au	Gold content	0.001	0.010	0	0.167
Nb	Niobium content	0.039	0.081	0	0.250
Hf	Hafnium content	0.018	0.055	0	0.250
Zr	Zirconium content	0.020	0.061	0	0.250
N	Nitrogen content	0.000	0.008	0	0.143
I	Iodine content	0.000	0.008	0	0.143
B	Boron content	0.001	0.011	0	0.154
W	Tungsten content	0.005	0.029	0	0.250
Ag	Silver content	0.001	0.010	0	0.167
Nd	Neodymium content	0.001	0.010	0	0.167
HV	Hardness	462.601	179.467	110	959.600

3 Preliminary Work for AAEG Training

3.1 HV mapping and bucketing from UTS

In the $\mathcal{S}$ dataset, we found six data points that have both HV and UTS target performance values. As shown in Figure S1a, there is a clear positive correlation between UTS and HV for these six data points, and their Pearson correlation coefficient is 0.86 Figure S1b. Therefore, we built a simple Linear Regression model to predict the $\mathcal{S}$ target performance HV based on the $\mathcal{S}$ performance UTS, and the R^2 value of the model reached 0.74 (Figure S1c). Since only UTS and EL labels are available

Table S2: Statistical information on the characteristics of the UTS and EL HEAs dataset $\mathcal{T}$.

Element	Description	mean	std	min	max
Al	Aluminum content	0.080	0.061	0.000	0.192
Co	Cobalt content	0.166	0.083	0.000	0.286
Cr	Chromium content	0.170	0.070	0.000	0.286
Fe	Iron content	0.211	0.082	0.000	0.670
Ni	Nickel content	0.255	0.079	0.000	0.482
Cu	Copper content	0.044	0.100	0.000	0.360
Mn	Manganese content	0.074	0.112	0.000	0.348
cr	Cold Rolling	17.348	30.167	0.000	90.000
TAN	Heat processing temperature	175.278	346.846	25.000	1200.000
tAN	Heat processing time	0.185	0.488	0.000	2.000
UTS	Ultimate tensile strength	778.362	301.394	368.000	1800.000
EL	Elongation	25.285	19.512	0.200	78.000

Table S3: Notations and descriptions

Notations	Description
$\mathcal{D}$	Domain
$\mathcal{X}$	Feature space
$P(X)$	Marginal probability distribution
$\mathcal{S}$	$\mathcal{S}$
$\mathcal{T}$	Taeget Domain
$\mathbf{L}$	Bucketed labels
N_c	Number of buckets
Y_{HV}	HV ground truth
Y_{UTS}	UTS ground truth
Y_{EL}	EL ground truth
$Y_c = \{Y_T,\ Y_F\}$	Y_c : Image real(Y_T)/fake(Y_F)label
$z \in \mathbb{R}^d$	Noise $\mathcal{N}(0, 1)$
$l \in 0, 1^{N_c+1}$	One-hot vector of the bucket label $\mathbf{L}$
$f_B(Y_{pred},\ Y_{true})$	Binary cross-entropy loss
$f_L(Y_{pred},\ Y_{true})$	Cross-entropy loss
$F:\ \mathbf{X} \mapsto \mathbb{R}^d$	Feature representation network
$C:\ \mathbb{R}^d \mapsto \mathbf{L}$	$\mathcal{S}$ bucketing classification network
$G:\ \{\mathbb{R}^d, z, l\} \mapsto \mathbf{X}'$	Generator network
$D:\ \{\mathbb{R}^d, \mathbf{X}, \mathbf{X}'\} \mapsto \{\mathbf{L},\ Y_c\}$	Discriminator network

in the $\mathcal{T}$, during the AAEG training, we can backpropagate the $\mathcal{T}$ label data in the D network. We mapped the UTS performance of $\mathcal{T}$ to HV performance of $\mathcal{S}$ using Linear Regression and bucketed it to provide the F network with a gradient of awareness of the $\mathcal{T}$ data. To verify the accuracy of the model's bucketing, we tested the model's bucketing accuracy based on the corresponding UTS and HV values in Ref.[17]. The confusion matrix of the model's bucketing is shown in Figure S1d, and the accuracy of this bucketing model reached 0.925, which proves that our bucketing accuracy for the HV performance in the $\mathcal{T}$ is sufficient to provide the correct cognitive gradient for the F network.

3.2 Alignment component feature

XenonPy [18] is a Python library that implements comprehensive machine learning tools for material informatics. XenonPy provides a rich set of tools for applying material informatics to various tasks, in which the descriptor generator can compute multiple types of numerical descriptors from the composition of the material. XenonPy's built-in descriptor generator can generate 58 element-level descriptor properties for 94 elements (from H to Pu) and uses seven statistics to obtain seven statistical features (see Table S5) for each element-level descriptor. Thus, by entering the chemical composition information of the material into the descriptor module, a matrix of element-level descriptor features ($58 * 7$ dimensions) was obtained. Ref. [18] obtained a dense feature matrix of size $56 * 9$ by discarding two economic statistical features and padding two zeros to each of the seven-dimensional statistical matrices.

Table S4: Alphabetically Ordered Abbreviations and Full Terms

**Abbreviation**	**Full Term**
DSMID	Dual-Strategy Materials Intelligent Design Framework
AAEG	Adversarial Domain Adaptive Embedding Generative Network
AMSEP	Automated Material Screening and Evaluation Pipeline
AC	As-Cast
BDA	Balanced Distribution Adaptation
CDAN	Conditional Adversarial Domain Adaptation
CORAL	Correlation Alignment
DAN	Deep Adaptation Network
DANN	Domain Adversarial Neural Network
EBSD	Electron backscatter diffraction
edRVFL	ensemble deep RVFL network
EHEA	Eutectic HEA
EL	elongation
GAN	Generative Adversarial Network
GND	geometrically necessary dislocations
GRL	Gradient Reversal Layer
HEA	High Entropy Alloy
HV	hardness
IM	Intermetallic
LLM	large language model
ML	machine learning
NSGA-II	Nondominated Sorting Genetic Algorithm
OOD	Out-of-Distribution
RF	Random Forest
RVFL	Random Vector Functional Link
SS	Solid Solution
TCA	Transfer Component Analysis
TrAdaBoost	Transfer AdaBoost
UTS	ultimate tensile strength
XGBoost	Extreme Gradient Boosting
XRD	X-ray diffractometer

We adopted a domain-knowledge-based feature mapping scheme from Ref. [19] to map the expanded information in Xenonpy to a 24×21 grayscale image, as shown in Figure S2. By this method, all chemical components can be mapped to the same high-dimensional space for feature alignment. This approach helps to avoid performance degradation of the model on the $\mathcal{T}$ due to differences in features between the $\mathcal{S}$ and $\mathcal{T}$, and enables the model to better capture the commonalities and differences between the $\mathcal{S}$ and $\mathcal{T}$, thus improving the model's generalization performance. This lays a solid foundation for the successful transfer of subsequent domain-adaptive models.

Table S5: XenonPy feature statistics calculation method [18]

Feature transformation method	Feature calculation formula
Weighted average	$f_{ave,i} = w_A^* f_{A,i} + w_B^* f_{B,i}$
Weighted variance	$f_{var,i} = w_A^* \left(f_{A,i} - f_{ave,i}\right)^2 + w_B^* \left(f_{B,i} - f_{ave,i}\right)^2$
Geometric mean	$f_{gmean,i} = \sqrt[w_A+w_B]{f_{A,i}^{w_A} * f_{V,i}^{w_B}}$
Harmonic mean	$f_{hmean,i} = \frac{w_A+w_B}{\frac{1}{f_{A,i}} * w_A + \frac{1}{f_{B,i}} * w_B}$
Max-pooling	$f_{max,i} = max\left(f_{A,i}, f_{B,i}\right)$
Min-pooling	$f_{min,i} = min\left(f_{A,i}, f_{B,i}\right)$
Weighted sum	$f_{ave,i} = w_A f_{A,i} + w_B f_{B,i}$

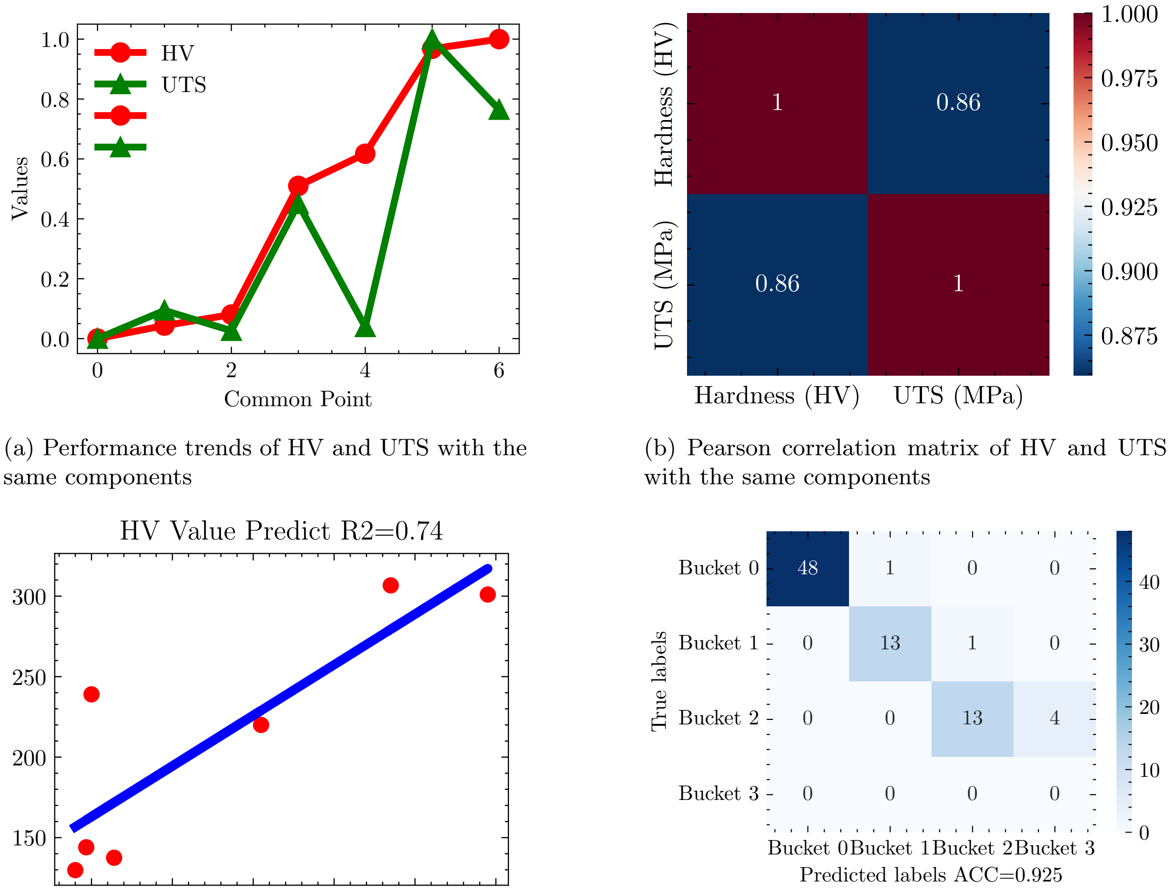

(a) Performance trends of HV and UTS with the same components

(b) Pearson correlation matrix of HV and UTS with the same components

(c) Linear regressor predicting HV based on UTS, with $R^2 = 0.74$

(d) Confusion matrix of HV values predicted by linear regressor based on UTS values from Ref. 17, with $Acc = 0.925$

Fig. S1: Process of mapping UTS to HV and bucketing.

4 Details of AAEG

4.1 Domain Adaptation Training Task

We compared regression and classification methods for domain adaptation training tasks. When embedding a regression task directly into the domain-adaptive model, the model faced significant challenges in converging and producing HEA composition characterization embeddings that met our requirements. The difficulties encountered in transferring the regression task include the following:

(1) When working with different data distributions, the boundaries between different categories tend to be more vivid, which indicates that the use of classification models is more suitable for differentiation purposes. Conversely, regression problems tend to relate to subtle differences that may lead to more intricate variations across various data distributions. Furthermore, the model has to predict not just continuous regression values but also reduce disparities between the source and the target domains, which puts significant strain on the model's convergence.

(2) Several formulas in domain adaptation are defined based on classification models, such as those founded upon important inter-domain distance and instance features. Nonetheless, such methods do not have the propensity to work on regression models.

(3) Typically, the training objective of domain adaptation models aims to minimize differences between the source domain and the target domain, which are often quantified in classification tasks. Since there are significant differences in nature between regression and classification tasks,

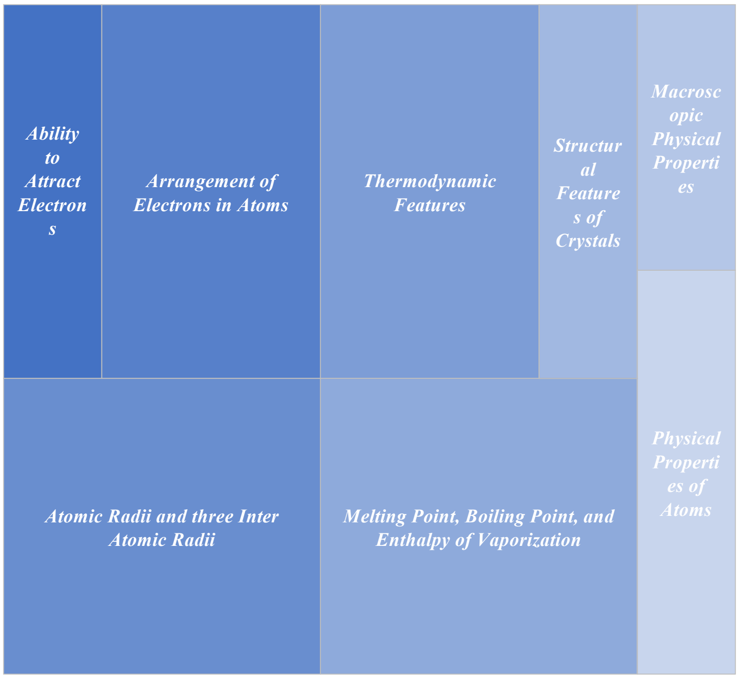

Fig. S2: Arrangement of compositional features [19]. The 56-dimensional features are divided into eight parts: (1) features related to the ability to attract electrons; (2) features related to the arrangement of electrons in atoms; (3) atomic radii and three inter atomic radii; (4) thermodynamic features; (5) structural features of crystals; (6) melting point, boiling point, and enthalpy of vaporization; (7) macroscopic physical properties of materials; and (8) physical properties of atoms.

adaptation methods used for classification tasks may not be appropriate for regression tasks, causing difficulties in achieving convergence.

Given the challenges in achieving convergence for regression tasks in domain adaptation models, we modified the neural network training task in the Deep Domain Adaptation method. Instead of predicting the regression value of the HV of HEAs, we switched to predicting the bucketing labels of HV values. This transformation process is detailed in § 3.1. By switching to a bucketing task, the composition feature representation network, F, can obtain a cognitive gradient of the data, enabling F to extract shallow and generalized features.

For the HV performance, the values are uniformly divided into four bins, ranging from 110 to 959.6 (see Table S1), with denotations of $N_C = 4$. To address the absence of HV performance bucketing in the target domain, we devised a simple Linear Regression model based on the comparison table of steel tensile strength and Vickers hardness [17]. This Linear Regression model categorizes the HV bucket label relevant to the UTS performance in the target domain, achieving an R^2 of 0.74(as described in § 3.1).

In the target domain, we use Linear Regression to map the UTS performance in the target domain to the HV performance in the source domain for bucketing during AAEG training. This mapped cognitive gradient is crucial for the F network to effectively analyze and learn from the target domain data.

4.2 Auxiliary Methods Used in the AAEG Framework

4.2.1 Generative Adversarial Network

Formally, traditional GAN involves two networks that are in competition: a discriminator D and a generator G. G and D engage in a minimax game theory where the goal of the generator network G is to generate realistic component feature images to mislead the discriminator network D, while

the goal of D is to accurately distinguish between the images generated by G and the real component feature images. This dynamic process between G and D forms a game process. To train GANs, the following optimization problem is solved iteratively [20].

$$\min_G \max_D V(D,G) = \mathbb{E}_{\boldsymbol{x}\sim p_{\text{data}}(\boldsymbol{x})}[\log D(\boldsymbol{x})] + \mathbb{E}_{\mathbf{z}\sim p_{\mathbf{z}}(\mathbf{z})}[\log(1 - D(G(\mathbf{z})))] \tag{1}$$

In practical settings, Eq. 1 may not provide sufficient gradient for improved learning performance by G. During the initial stages of G's training when its performance is unsatisfactory, D can confidently reject generated samples solely produced by G using noise vector z because they are distinguishable from the training data. To address this issue, Auxiliary Classifier GAN (AC-GAN) [13, 21] was employed. AC-GAN introduces the bucketing labels $\boldsymbol{L}$ in the G's input one-hot encoded along with the embeddings extracted from F. Additionally, to effectively reduce the domain differences between the source and target domains through adversarial training between F and D, the input connection of the embeddings extracted by F were fed into D. Refer to Algorithm **??** for more detailed information on how the networks D and G are updated.

We employ GAN for domain adaptation at the pixel level for the following reasons: (1) In the HEAs dataset, certain properties have a limited amount of data due to measurement difficulties. The use of GANs can generate realistic target domain data, thereby increasing data utilization and enabling improved utilization of the available target domain data. (2) GANs have a mechanism where the generator and discriminator are adversarial to learn data distribution, enabling the generator to generate more realistic target domain data, while the discriminator is tasked with accurately discriminating real data from generated data. Meanwhile, this adversarial training mechanism can encourage the model to learn more robust feature representations of HEAs, thereby enhancing the model's generalization ability.

4.2.2 Deep CORAL Loss

The CORAL loss utilizes a linear transformation technique to align the second-order statistical characteristics in the source and target domain distributions, making it a powerful tool for unsupervised domain adaptation. In contrast, Deep CORAL loss utilizes a non-linear transformation method that can be easily incorporated into CNN or FNN models. The distance between the second-order statistics (covariance) of the source and target features defines the CORAL loss as Eq. 2 [22].

$$\ell_{CORAL} = \frac{1}{4d^2}\left\|C_{\mathcal{S}} - C_{\mathcal{T}}\right\|_F^2 \tag{2}$$

where $\|\cdot\|_F^2$ denotes the Frobenius norm of the matrix F, and $C_{\mathcal{S}}$ and $C_{\mathcal{T}}$ represent the covariance matrices of the source and target data, respectively. The variable d denotes the dimensionality of the feature space and is used to normalize the result of the Frobenius norm.

$$\begin{aligned} C_{\mathcal{S}} &= \frac{1}{n_{\mathcal{S}}-1}\left(D_{\mathcal{S}}^\top D_{\mathcal{S}} - \frac{1}{n_{\mathcal{S}}}\left(\mathbf{1}^\top D_{\mathcal{S}}\right)^\top\left(\mathbf{1}^\top D_{\mathcal{S}}\right)\right) \\ C_{\mathcal{T}} &= \frac{1}{n_{\mathcal{T}}-1}\left(D_{\mathcal{T}}^\top D_{\mathcal{T}} - \frac{1}{n_{\mathcal{T}}}\left(\mathbf{1}^\top D_{\mathcal{T}}\right)^\top\left(\mathbf{1}^\top D_{\mathcal{T}}\right)\right) \end{aligned} \tag{3}$$

where $n_{\mathcal{S}}$ and $n_{\mathcal{T}}$ respectively denote the number of data samples in the source and target domains, and the gradient of the Deep CORAL Loss is defined as Eq. 4.

$$\begin{aligned} \frac{\partial l_{\text{CORAL}}}{\partial D_{\mathcal{S}}^{ij}} &= \frac{1}{d^2\left(n_{\mathcal{S}}-1\right)}\left(\left(D_{\mathcal{S}}^T - \frac{1}{n_{\mathcal{S}}}\left(\mathbf{1}^T D_{\mathcal{S}}\right)^T \mathbf{1}^T\right)^T \left(C_{\mathcal{S}} - C_{\mathcal{T}}\right)\right)^{ij} \\ \frac{\partial l_{\text{CORAL}}}{\partial D_{\mathcal{T}}^{ij}} &= -\frac{1}{d^2\left(n_{\mathcal{T}}-1\right)}\left(\left(D_{\mathcal{T}}^T - \frac{1}{n_{\mathcal{T}}}\left(\mathbf{1}^T D_{\mathcal{T}}\right)^T \mathbf{1}^T\right)^T \left(C_{\mathcal{S}} - C_{\mathcal{T}}\right)\right)^{ij} \end{aligned} \tag{4}$$

Adversarial training of the D and F networks leads to F's component representational embeddings, becoming progressively more discernible by D. Furthermore, adversarial training of G on par with F often hinges on sufficient data and a great number of iterations. Subsequently, the effectiveness of the extracted features in the target domain may suffer. As a result, in this paper, we have chosen to integrate the deep CORAL loss after the F network. The detailed reasons include: (1) The CORAL Loss characterizes the learning of representations by differentiating the correlation between covariances, fundamentally pursuing commonalities between different domains. Its principal advantage lies in its simplicity of calculation and differentiability, which permits the updating of network parameters through back-propagation. (2) The Deep CORAL Loss can be employed to address the domain shift between sets $\mathcal{S}$ and $\mathcal{T}$ by increasing the difficulty of distinguishing between the distributions D of the two sets. Additionally, it serves to amplify the representation of information from $\mathcal{T}$ within the embedding vectors extracted by F, thus enhancing accuracy in regression tasks specific to $\mathcal{T}$.

4.2.3 Gradient Reversal Layer

During training, to achieve domain-invariant features, we aim to determine feature mapping parameters, denoted as θ_F, that maximize the domain classifier loss in D while ensuring that data from the two domains share similar feature distributions. Similarly, we seek to determine domain classifier parameters, denoted as θ_D, that minimize the domain classifier loss in D. To update F using gradients derived from domain classifier losses in D, we incorporate the gradient reversal layer (GRL) as Eq. 5 [23.]

$$\theta_F \leftarrow \theta_F - \mu \left(\frac{\partial L'}{\partial \theta_F} - \lambda \frac{\partial L_{\text{data}}}{\partial \theta_F} \right) \tag{5}$$

L_{data} denotes the domain classification loss acquired by F from D, while L' represents other losses obtained by F from C, G, and D, with μ denoting the learning rate and λ representing the parameter that balances the objectives of reducing domain shift and improving bucket accuracy in F. To achieve an adversarial effect, GRL multiplies the domain classification loss received by the layer by the negative parameter $(-\lambda)$, thereby reversing the training objectives between the layers before and after the GRL.

The introduction of GRL into the F and D networks serves to achieve an adversarial effect, primarily for two reasons: (1) GRL achieves domain adaptation at the feature level, whereas GAN achieves adaptation at the pixel level. As a result, GRL is better suited than GAN for establishing the adversarial relationship between the representation network F and the discriminative network D. (2) Using GRL, the gradients are reversed to impose opposing objectives on the two networks. F's goal is to generate component representations that share embedding distributions as closely as possible for the source and target domains, whereas the D network aims to improve discrimination between the imbalanced aspects of the two domains.

4.2.4 Ensemble deep RVFL

The output layer of the RVFL (Random Vector Functional Link) model [24] receives a fusion of non-linear features generated from the hidden layer $\boldsymbol{H}$) and the original input features ($\boldsymbol{X}$). Due to the fact that the parameters in the hidden layer are randomly-generated and immutably retained to their initial values during the training stage, the only essential objective is to obtain the optimized output weigh β_s. The resultant optimization problem is mathematically represented as Eq. 6.

$$\min_{\boldsymbol{\beta}_s} \|\mathbf{D}\boldsymbol{\beta}_s - Y\|^2 + \lambda \|\boldsymbol{\beta}_s\|^2 \tag{6}$$

where $\mathbf{D} = [\boldsymbol{H}\ \boldsymbol{X}]$ is the concatenation of the output of the hidden layer, denoted as $\boldsymbol{H}$, and original features, denoted as $\boldsymbol{X}$. The parameter $\lambda \|\boldsymbol{\beta}_s\|^2$ is used for regularizing the model, where Y represents the target vector. The pivotal idea behind RVFL is to leverage the enhanced non-linear features learned by the hidden layer to increase the generalization capability of the model for the original data.

The dRVFL (Deep Random Vector Functional Link) model [25] is a type of RVFL network that serves as an extension in the fields of representation or deep learning. In dRVFL, the input to each layer is the output from the previous layer, and as such, each layer creates an internal representation of the input data Eq. 7.

$$\begin{aligned} \mathbf{H}^{(1)} &= g\left(\mathbf{X}\mathbf{W}^{(1)}\right) \\ \mathbf{H}^{(L)} &= g\left(\mathbf{H}^{(L-1)}\mathbf{W}^{(L)}\right) \\ \mathbf{D} &= \left[\mathbf{H}^{(1)}\mathbf{H}^{(2)}\ldots\mathbf{H}^{(L-1)}\mathbf{H}^{(L)}\mathbf{X}\right] \end{aligned} \tag{7}$$

where non-linear activation function is denoted by $g(\cdot)$, where $\mathbf{X}\mathbf{W}^{(n)}$ represents the randomly generated parameters of layer n.

The edRVFL employs abundant intermediate characteristics to make the ultimate decision, which distinguishes it from dRVFL. At each hidden layer, the input comprises non-linearly transformed features from the previous layer Eq. 8 (similar to dRVFL) and the original input features from standard RVFL (which are directly connected).

$$\mathbf{H}^{(L)} = g\left(\left[\mathbf{H}^{(L-1)}\mathbf{X}\right]\mathbf{W}^{(L)}\right) \tag{8}$$

The edRVFL is regarded as a hybrid of dRVFL and ensemble learning. Unlike conventional ensemble methods that demand the independent training of numerous models from the beginning, edRVFL is produced by training a single dRVFL network. Thus, edRVFL incurs only a small overhead while achieving higher accuracy compared to training a single dRVFL model. Our final regression predictor is edRVFL, chosen for two primary reasons: (1) The training of edRVFL is faster, cheaper, and does not need backpropagation for parameter updates, unlike traditional neural network models. Additionally, edRVFL employs ensemble learning strategy to mitigate overfitting and variance, thus improving model generalizability. (2) In the regression task, the input consists of embeddings obtained from the F neural network. As a neural network-based approach, edRVFL can efficiently handle high-dimensional, nonlinear, and complex data without using backpropagation to update parameters. Furthermore, edRVFL demonstrates superior adaptability to embeddings compared to tree or support vector machine models.

4.3 Network architecture details

The specific parameters of each module network in the AAEG network are as follows:

- $F : C(64, 3, 3) - P(2, 2) - C(128, 3, 1) - P(2, 2) - L(128, 64)$
- $C : L(64, 32) - LeakReLU(0.2) - L(32, N_c)$
- $G : C_T(64, 2, 1, 0) - B(64) - C_T(32, 4, 2, 1) - B(64) - C_T(16, (4, 3), 2, 1) - B(64) - C_T(1, 4, 3, 1) - Tanh()$
- $D : C(64, 3, 3) - P(2, 2) - C(128, 3, 1) - P(2, 2) - L(128, 64)$
  * $-L(64, 32) - LeakReLU(0.2) - L(32, N_c)$
  * $-L(64, 32) - LeakReLU(0.2) - L(32, 1) - Sigmoid()$

where $C(c, k, s)$ represents a standard convolutional layer with $k * k$ filters, c channels, and s stride; $P(k, s)$ represents a Maxpooling layer with $k * k$ filter size and s stride; $L(i, o)$ represents a fully connected (linear) layer that maps features from i dimensions to o dimensions; $C_T(c, k, s, p)$ represents

a transposed convolutional layer with $k * k$ filters, c channels, s stride, and p padding size; $B(c)$ represents a Batch Normalization layer with c channels.

We implemented a 2-layer CNN network in the F network to extract embeddings with improved ability to generalize in the $\mathcal{T}$. The shallow network helps extract common features from images in both the $\mathcal{S}$ and $\mathcal{T}$, which are composed of images arranged according to the same rules. The first CNN layer is designed to extract $3 * 3$ window information based on Ref. [19].

4.4 Training and Testing Process for AAEG

Algorithm S1 shows the the training and testing process of AAEG.

4.5 Evaluation Metrics and Loss Function Setup

The Adversarial Domain Adaptive Embedding Generative Network (AAEG) successfully employed transfer learning across various datasets of HEAs to generate embeddings that exhibit superior predictability by a regressor, thereby validating the model's effectiveness. The regression task involved the use of the coefficient of determination R^2 (Eq.9), mean squared error (MSE, Eq.10), and mean absolute percentage error (MAPE, Eq.11).

$$R^2 = \frac{\sum (\hat{y}_i - \bar{y})^2}{\sum (y_i - \bar{y})^2} = 1 - \frac{\sum (y_i - \hat{y})^2}{\sum (y_i - \bar{y})^2} \tag{9}$$

$$\mathrm{MSE}(y, \hat{y}) = \frac{\sum_{\mathrm{i}=1}^{n} (\hat{y}_i - y_i)^2}{n} \tag{10}$$

$$\mathrm{MAPE} = \frac{\sum_{\mathrm{i}=1}^{n} \left(\frac{\hat{y}_i - y_i}{\hat{y}_i}\right)^2}{n} \tag{11}$$

During AAEG training, we utilized cross-entropy loss (Eq.12) and binary cross-entropy loss (Eq.13). The former was employed for bucket training of HV target performance, while the latter was leveraged by the D network to distinguish between feature images of the composition and generated images, as well as between images from the source and target domains.

$$f_L(y, \hat{y}) = -\sum_{k}^{N} \hat{y}_k \log(y_k) \tag{12}$$

$$f_B(y, \hat{y}) = -\frac{1}{N} \sum_{i=1}^{N} \hat{y}_i \cdot \log(y_i) + (1 - \hat{y}_i) \cdot \log(1 - y_i) \tag{13}$$

4.6 Training Losses of AAEG

This section presents the loss curves observed during the training process of the AAEG. The plotted losses include the F, C, D, G network loss, which collectively contribute to the effectiveness of the proposed method.

The G loss exhibits significant fluctuations during the training process. This behavior is characteristic of GAN networks, where the generator (G) and discriminator (D) are engaged in a continuous adversarial game. As the generator improves, the discriminator adapts to better distinguish between real and generated samples, causing oscillations in the generator's loss. These fluctuations are indicative of the dynamic balance being struck between the generator and discriminator, and they are essential for achieving high-quality generative performance.

Algorithm S1 Training and testing processes for AAEG

function FEATUREMAPPING($\mathcal{S}$, $\mathcal{T}$) ▷ $\mathcal{S}$ and $\mathcal{T}$ Feature Alignment
    Map the component features of the $\mathcal{S}$ and $\mathcal{T}$ to grayscale images with the method [19].
    Place the obtained component mapping images into their corresponding domains $\mathcal{D}$.
end function
function BUCKETLABELING($\mathcal{S}$, $\mathcal{T}$) ▷ Segmentation and Bucket Labeling
    Segment the Y_{HV} performance into distinct categories and assign them to appropriate buckets, denoted as $\mathbf{L}_{\mathcal{S}}$.
    Build a linear regression model based on the common data of Y_{HV} and Y_{UTS} in $\mathcal{S}$.
    Map the Y_{UTS} values in domain $\mathcal{T}$ to corresponding Y_{HV} values and categorize them into designated buckets, represented by $\mathbf{L}_{\mathcal{T}}$.
    return Bucket Labels $\mathbf{L}_{\mathcal{S}}$ and $\mathbf{L}_{\mathcal{T}}$.
end function
function TRAINING($\mathcal{S}$, $\mathcal{T}$, $\mathbf{L}_{\mathcal{S}}$, $\mathbf{L}_{\mathcal{T}}$) ▷ Adversarial Training Representation Network F
    Randomly Initialize models parameters θ_F, θ_C, θ_G, θ_D.
    Perform $\mathcal{S}$ bucketing task with network C.
    Update θ_F and θ_C using the computed cognitive gradient ∇_C.
    Perform $\mathcal{S}$ and $\mathcal{T}$ bucketing task, $\mathcal{S}$ and $\mathcal{T}$ classification task, and real/fake image discrimination task using network D.
    Compute the gradients ∇_D based on the tasks performed by network D.
    Update the θ_F, θ_G, θ_D using the computed gradients ∇_D.
    Compute the embeddings of $\mathcal{S}$ and $\mathcal{T}$ using network F.
    Compute the ∇_{CORAL} based on the computed embeddings.
    Update the θ_F using the ∇_{CORAL}.
    return Trained θ_F.
end function
function TESTING($\mathcal{S}$, $\mathcal{T}$, θ_F) ▷ Test Regression Model with Representation Vectors
    Input the components of the $\mathcal{S}$ and $\mathcal{T}$ into the F network to obtain component representation embeddings.
    Concatenate the component representation embeddings with other process features to obtain the transferred features.
    Train the $edRVFL$ regression model to obtain a high-precision performance prediction model for HEAs.
    return Trained $edRVFL$ model $\mathcal{M}$.
end function
function PARETOFRONTEXTRAPOLATION($\mathcal{M}$) ▷ Pareto front extrapolation with NSGA-II
    Initialize constraint function $g(x)$, population size N, maximum number of iterations T.
    Apply NSGA-II algorithm for UTS and EL objectives with $\mathcal{M}$.
    return UTS and EL pareto front set P.
end function
function EXPERIMENTALVALIDATION(P) ▷ Validate HEAs Experimentally
    Screen test points and validate through experimental preparation.
    return HEAs meeting performance requirements.
end function
Input: $\mathcal{S}$, $\mathcal{T}$, HV, UTS ▷ Main
FeatureMappingImages ← FEATUREMAPPING($\mathcal{S}$, $\mathcal{T}$)
Bucket Labels $\mathbf{L}_{\mathcal{S}}$ and $\mathbf{L}_{\mathcal{T}}$ ← BUCKETLABELING($\mathcal{S}$, $\mathcal{T}$)
ModelParameters θ_F ← TRAINING($\mathcal{S}$, $\mathcal{T}$, $\mathbf{L}_{\mathcal{S}}$, $\mathbf{L}_{\mathcal{T}}$)
edRVFLModel $\mathcal{M}$ ← TESTING($\mathcal{S}$, $\mathcal{T}$, θ_F)
ParetoFrontSet P ← PARETOFRONTEXTRAPOLATION($\mathcal{M}$)
Output: ValidatedHEAs ← EXPERIMENTALVALIDATION(P)

4.7 Visualization of Domain Adaptation with AAEG.

The source domain dataset $\mathcal{S}$ contains 23 dimensions of component information, while the target domain dataset $\mathcal{T}$ consists of only 7 dimensions of component features, which are a subset of $\mathcal{S}$'s component features. The domain shift between the two becomes more pronounced when components that are only present in $\mathcal{S}$, such as silicon (Si), appear, making it difficult to transfer the component

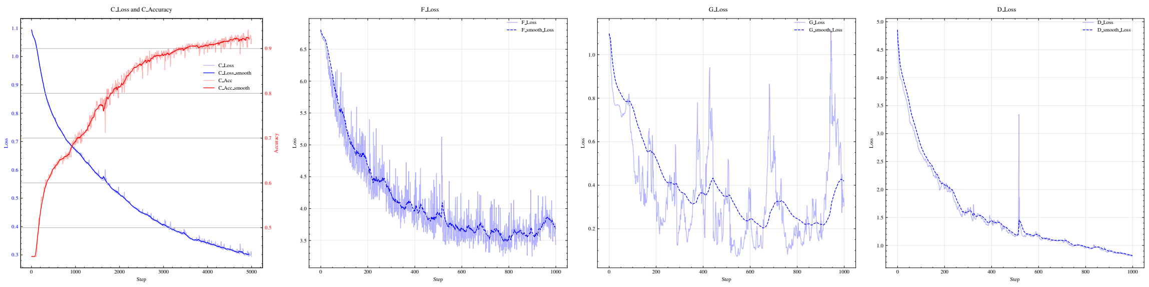

(a) C Loss and Accuracy (b) F Loss (c) G Loss (d) D Loss

Fig. S3: Training losses of AAEG

representation model built on $\mathcal{S}$ to $\mathcal{T}$. To visualize the performance of the component representation embedding generated by the AAEG network in domain adaptation, we used t-SNE [26] to visualize the component feature vectors before and after transfer (as shown in Figure S4). The figure shows that the original source domain (blue dots) and the target domain (red dots) are concentrated in the lower right and upper left corners, respectively, indicating a significant domain shift. After the AAEG transfer, the distribution of the embedding vectors becomes more uniform, with a smaller distribution difference between the source and target domains.

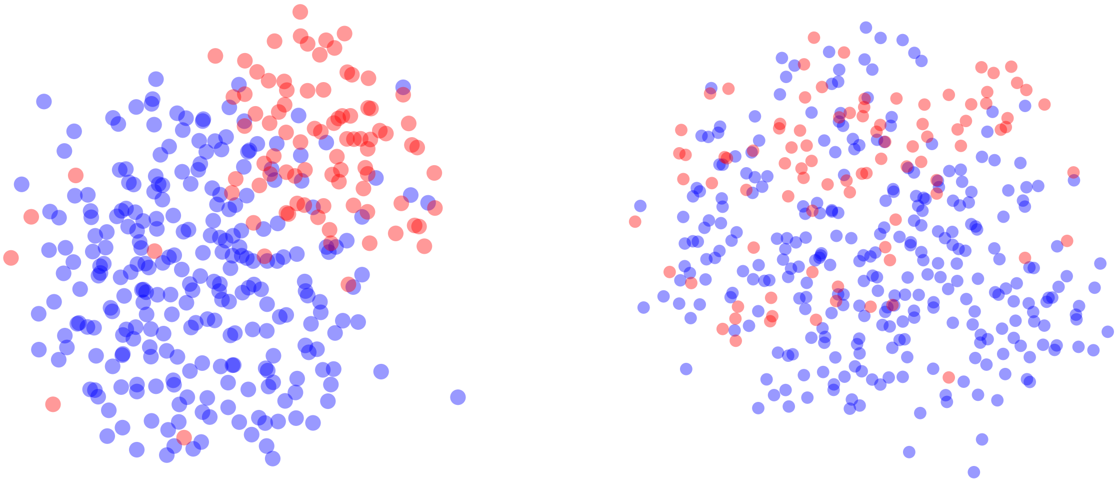

(a) Non adapted (b) Adapted

Fig. S4: TSNE visualization of $\mathcal{S} \rightarrow \mathcal{T}$ adaptation. In (a), the source data shown in blue is mainly distributed in the lower left corner but target data shown in red is mainly distributed in the upper right corner, and there is a significant shift in the distribution of both. On applying the proposed approach, as shown in (b), source and target data are more evenly and closely distributed.

4.8 Ablation Study

To reveal the effectiveness of the AAEG network structure and determine its optimal architectural configuration, this study carried out ablation experiments whose outcomes are showcased in Table S6. The baseline is the model with only the AEEG framework, which lacks the losses generated by Deep CORAL and the residual connections between F and D.

Therefore, from Table S6, augmenting the CORAL loss and GRL (Residual) can significantly improve the baseline model's predictive accuracy, especially for UTS performance, where the inclusion of GRL (Residual) boosted accuracy by 15%, as observed in the ablation experiments.

Table S6: R^2 results of ablation experiments. Baseline is the GAN-only AAEG, *+ CORAL* refers to the addition of Deep CORAL Loss at the F output, *+ GRL (Residual)* refers to the establishment of residual connections between F and D and the addition of a GRL layer.

Models	HV ($\mathcal{S} \to \mathcal{T}$)	UTS ($\mathcal{S} \to \mathcal{T}$)	EL ($\mathcal{S} \to \mathcal{T}$)
Baseline	0.815	0.645	0.789
+ CORAL	0.84	0.704	0.809
+ GRL (Residual)	0.866	0.803	0.812
AAEG	**0.919**	**0.804**	**0.849**

Here, the effectiveness of the residual connection and deep CORAL loss in yielding positive outcomes can be elucidated through the following essential reasons:

(1) Residual connections were incorporated to enhance the convergence speed and elevate the bucketing accuracy of the D network, which accepts inputs from both domains, in contrast to C. Additionally, the limited amount of data resulted in G's poor convergence compared to D, preventing G from misleading D, and restricting F to rely only on gradients from G, making it incapable of effectively establishing the fundamental links between source and target domain features. Furthermore, the exceptional performance of DANN prompted an exploration of the adversarial interplay between F and D, instigated by GRL, which has the potential to facilitate the extraction of embeddings by F.

(2) The Deep CORAL Loss implemented at the output of the F network plays a crucial role in diminishing domain shift. This is achieved by minimizing the distance of the covariance matrix between features from the source and target domains, thereby aligning the feature space. Unlike data-driven and black box Deep Domain Adaptation methods, Deep CORAL Loss employs nonlinear transformations and a formula to calculate the distance of inter-domain covariance to reduce domain shift. Furthermore, it can notably improve transfer quality even on limited datasets.

4.9 Correlation analysis between generated images and target performance

The main objective of the generative network G has shifted from generating as realistic images as possible to providing gradients that reduce domain shift to the representation network F. To verify if G can provide the correct gradients to F for component feature representation, we conducted a pearson correlation matrix analysis between the generated images and the $\mathcal{T}$ performance Figure S5. The features in green circles show a positive correlation with the target performance, while those in yellow circles exhibit a negative correlation. The results were compared with the knowledge in the field of HEAs.

(1) UTS and EL performance exhibit a significant negative correlation in the domain knowledge. The correlation analysis matrix shows that their correlation coefficient is -0.48 in this dataset, which is consistent with the expectations from domain knowledge.

(2) The absolute value of the impact of features in the circle on target performance is above 0.3, which is considered a relatively high correlation among the $24 * 21$ dimensional features. It also conforms to the first rule, where the same feature exhibits a positive correlation with one target performance and a negative correlation with the other target performance. Here are some feature explanations that are highly related to domain knowledge:
   - The mean atomic volume feature shows a positive correlation of 0.56 with UTS and a negative correlation of -0.36 with EL. As the average atomic volume of the components increases, the lattice constant also increases. This makes it more difficult for HEAs to deform and slip, thus resulting in higher tensile strength. At the same time, the increase in lattice constant leads to larger spacing between atoms, which reduces the elongation of HEAs [27].

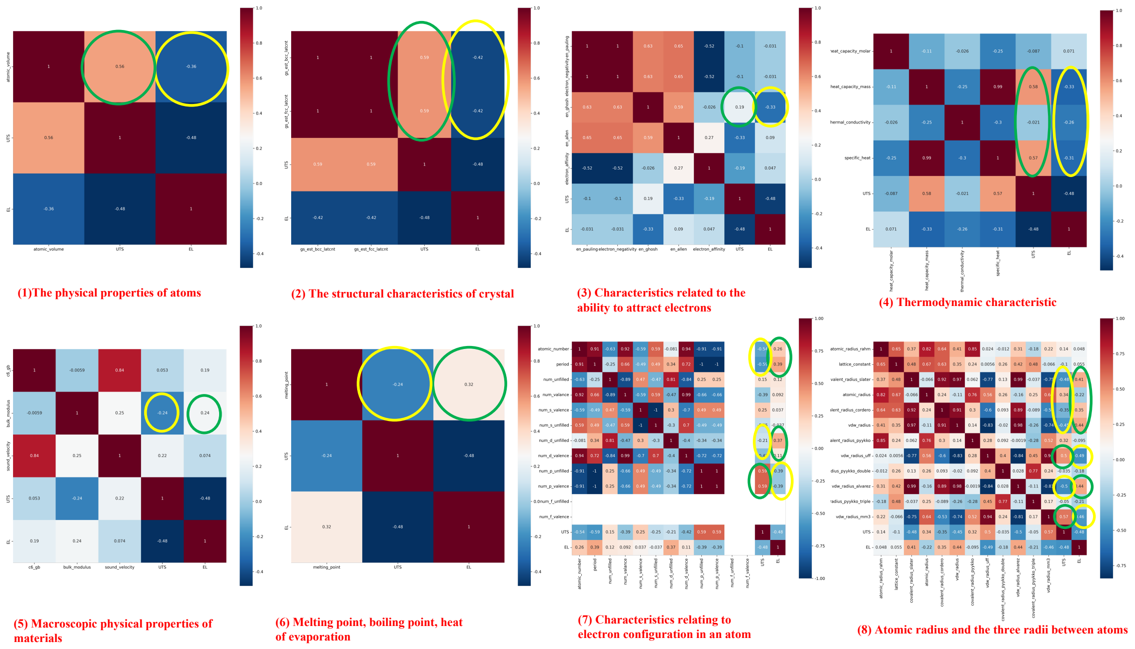

(a) Correlation analysis of average features in generated images with $\mathcal{T}$ UTS and EL performance.

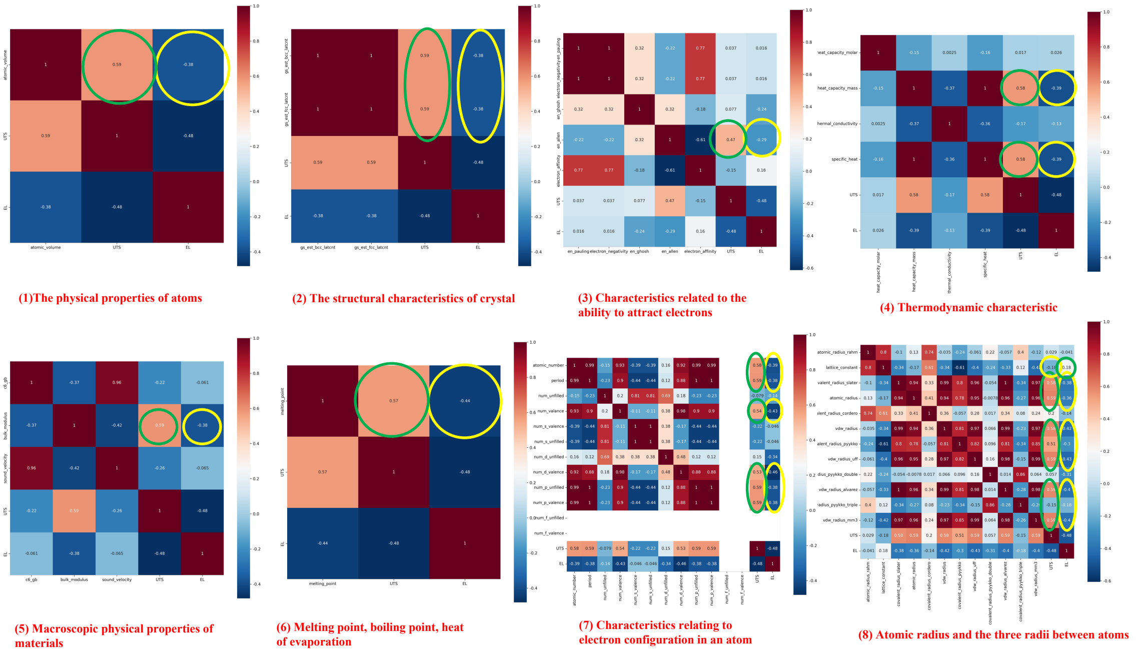

(b) Correlation analysis of variance features in generated images with $\mathcal{T}$ UTS and EL performance.

Fig. S5: Correlation analysis of partial features in generated images from $GNetwork$ with target performance, where values closer to 1 indicate higher positive correlation and values closer to -1 indicate higher negative correlation. The features in the green circle denote a large positive correlation, while those in the yellow circle denote a large negative correlation.

- The mean atomic number feature exhibits a negative correlation of -0.54 with UTS and a positive correlation of 0.25 with EL. This may be due to the addition of Al elements in the dataset, which could lead to a decrease in UTS, and the G network learns this pattern from the generated images. As the average atomic number of HEAs increases, it typically means that the alloy contains more highly electronegative elements such as chromium(Cr) and molybdenum(Mo), which increase the material's plastic deformation and thus enhance elongation. At the same time, the presence of highly electronegative elements causes the bonding between

material atoms to be more biased towards covalent bonds rather than metallic bonds, which weakens the atomic bonding and may therefore decrease the tensile strength [28].

- The mean covalent radius slater feature shows a negative correlation of -0.48 with UTS and a positive correlation of 0.42 with EL. The increase in the average covalent radius in HEAs' compositions can decrease the material's tensile strength. This is because a larger covalent radius usually implies weaker chemical bonds between elements, which can easily break under external stress and lower the material's tensile strength. However, an increase in the covalent radius does not necessarily result in worse elongation of the material. In some cases, an increased covalent radius can provide more slip paths for dislocations, thereby increasing the material's elongation [29].
- The mean period feature shows a negative correlation of -0.59 with UTS and a positive correlation of 0.39 with EL. This is because the periodic increase usually indicates the presence of more large-sized atoms in the material, which usually have stronger chemical reactivity and higher electronegativity, thereby increasing the elongation of the material [28].

In fact, the performance of HEAs is influenced by various complex factors, such as alloy composition, microstructure, types of chemical bonds, and so on. Therefore, when designing and optimizing HEAs, it is necessary to consider and balance the interactions of multiple factors. The considerations we have proposed above are only a partial reflection for reference.

5 Hyperparameter configuration

5.1 AAEG parameters setting

Table S7 shows a list of AAEG model hyperparameters and their values used in experiment. It is noteworthy that the learning rate used in the G network is 10 times that of other networks. In order to make the CORAL loss comparable to the cross-entropy and binary cross-entropy losses, we multiply it by the parameter λ. The value of λ is closely tied to the current training iteration, with a larger value indicating that the CORAL loss gradually dominates during updates towards the end of training. During the early stages of training, the goal is to build sensitivity of component representation embeddings to the bucketing task. In later stages, the main objective is to reduce domain discrepancy between the $\mathcal{S}$ and $\mathcal{T}$.

Table S7: AAEG model hyperparameters setting

Parameter	Setting value
imageSize	(24, 21)
Noisy z size	512
Noisy z std	0.19
Noisy z mean	0.44
batchSize	512
Embedding size	64
n_epochs	5000
optimizerF, optimizerC, optimizerD	Adam(lr=0.0008, betas=(0.8, 0.999))
optimizerG	Adam(lr=0.008, betas=(0.8, 0.999))
lr_decay_rate	0.0001
α	0.1
β	1.5
λ	$\frac{epoch}{n_{epochs}} * 1e6$

5.2 NSGA-II parameters setting

NSGA-II can be used to solve multi-objective optimization problems [30]. We use the NSGA-II algorithm to maximize both the UTS and EL metrics simultaneously, with specific parameter settings shown in Table S8.

Table S8: NSGA-II algorithm hyperparameters setting

Parameter	Setting value
objective function	The trained edRVFL model.
constraint	{TAN == 25 and tAN == 0} and {TAN != 25 and tAN !=0}
variable range	Table S9
population size	256
maximum generation	200
crossover probability	0.9
mutation probability	0.1
crossover distribution index	20
mutation distribution index	20
Initialization method	Random initialization
Termination criteria	{No improvement in 10 generations} or {Average UTS >1300 and EL >20}
Selection operator	Tournament selection
Replacement operator	Crowding distance replacement
Fitness assignment method	Non-dominated sorting
Crowding distance assignment method	Crowding distance

Table S9: The range of each feature genetic pool of NSGA-II algorithm, $[s, e]$ means the range of values taken from s to e, $x, ...$ means taking one of these values.

Features	Genetic pools
Al	[0.25, 4]
Co, Fe, Cr	{1}
Ni	[0.25, 4]
Cu	[0, 2]
Mn	[0, 2]
cr	{0}
TAN	{25, 650, 800, 900, 1000, 1200}
tAN	{0, 0.5, 1}

6 Details of AMSEP

6.1 Key Search Points and Evaluation Rules

In this section, we delineate the key points for executing Web and Document searches as advised by experts. Rather than requiring specific, definitive rules, the process necessitates only the essential keywords for the query, mimicking the manner in which one interacts with large language models (LLMs) or conducts online searches for pertinent information. This method significantly reduces the barriers to evaluating experimental schemes. Additionally, the LLM facilitates the subsequent screening process by querying, transforming, and evaluating, which markedly decreases the requirement for human labor. Below, we present the key points. These can be adapted for various tasks to yield diverse screening criteria and assessment outcomes.

Building upon the Key Search Points previously outlined, GPT-4 automates the retrieval of relevant knowledge from Vector Knowledge Base and the Web, subsequently synthesizing the information and translating it into clear assessment evaluation rules for experimental candidate schemes.

Algorithm S2 Key Search Points

- Eutectic high entropy alloys composition including Al, Co, Cr, Fe, Ni with optional elements Mn, Cu.
- Incorporating too many elements in a high entropy alloy affects the casting process, particularly in terms of remelting and elemental segregation.
- The specific issues and complications that arise from including Mn in alloy compositions during the casting process.
- Effects of cold rolling, heat treatment temperature, and time on eutectic high entropy alloys.
- The necessity of heat treatment in alloy production, its impact on production costs, and how avoiding it aligns with green manufacturing principles.
- Characteristics of eutectic high entropy alloys with high UTS and EL.
- The formation of solid solutions governed by the Hume-Rothery rules.

Algorithm S3 Evaluation Rules

- **Elemental Selection:** Choose elements that form a single-phase solid solution or a simple eutectic system. The elements Al, Co, Cr, Fe, Ni are commonly used in EHEAs due to their compatibility and ability to form stable high-entropy mixtures.
- **Optional Elements Effect:** Cu enhances corrosion resistance and conductivity but may lower thermal stability. Mn alters fluidity and solidification, risking casting defects and phase instability, leading to unwanted phases or microstructures.
- **Atomic Size Difference:** The atomic radii of the constituent elements should differ by a margin of 5% to 15%, a range that assists in maintaining a stable solid solution while simultaneously minimizing lattice distortion. See Microstructure evolution and mechanical properties of a novel CrNbTiZrAlx ($0.25 \leq x \leq 1.25$) eutectic refractory high-entropy alloy and Hume-Rothery rules for reference.
- **Valence Electron Concentration (VEC):** The VEC should be in a range that favors the formation of desired phases (like FCC, BCC, or HCP). The phase stability in high entropy alloys is often influenced by the electron concentration.
- **Mixing Enthalpy:** Select elements with negative or low positive mixing enthalpies to promote solid solution formation and reduce the tendency for intermetallic compound formation. See Seaweed eutectic-dendritic solidification pattern in a CoCrFeNiMnPd eutectic high-entropy alloy for reference.
- **Advancing Green Manufacturing:** To maintain the intrinsic strength and castability of eutectic high entropy alloys with minimal heat treatment and post-processing, adherence to green manufacturing principles is essential, streamlining the production process.
- **Phase Diagram Analysis:** Identify eutectic points where a simple solid solution or a combination of phases (like FCC+B2) can form. Look for compositions that avoid brittle intermetallic phases. Select compositions near eutectic points that favor the formation of desired solid solution phases.
- **Weighted Average Physical Properties:** Consider the weighted average of physical properties like melting point, density, and elasticity of the constituent elements.

6.2 Experimental Candidate Scheme Evaluation Results

Utilizing the NSGA-II algorithm, 1030 high-performance HEA experimental candidate scheme were identified. Leveraging the objectives we defined and its existing knowledge on HEAs, GPT-4 conducted an initial screening from a pool of 1,030 data points, yielding 362 prospective experimental schemes for further evaluation. This preliminary step was implemented to optimize resource allocation for subsequent individual assessments. We then furnished GPT-4 with the necessary computational data to evaluate the characteristics as prescribed by the criteria in Algorithm S3. Following this, GPT-4 individually scored each candidate scheme, also providing rationales for its assessments to aid experts in identifying the most promising experimental candidates. Due to display limitations for attachments, only the top three ranked schemes are provided here Table S10. For an in-depth understanding of the workflow, code, and logs of GPT-4 interactions, please refer to https://github.com/yuyouyu32/MaterialFilter

Table S10: Summary of GPT-4 Evaluation Results and Corresponding Detailed Reasons Presented by Index Number

HEAs	Processing			Predictive properties		Evaluation	
	cr	TAN	tAN	UTS	EL	Score	Reason
**Al14Co19Cr19Fe19Ni29**	**0**	**25**	**0**	**1007.91**	**17.00**	**0.85**	①
Al28Co11Cr11Fe11Ni39	0	800	1	1166.49	17.75	0.85	②
Al14Co19Cr19Fe19Ni19Mn9	0	800	1	1056.24	12.95	0.85	③

① **Al14Co19Cr19Fe19Ni29**:The component selection aligns with the elemental selection rule, utilizing common EHEA elements (Al, Co, Cr, Fe, Ni) that are compatible and known for forming stable high-entropy mixtures, which is favorable. The atomic size difference of 4.19% is within the optimal range (5%-15%), slightly missing the lower bound, indicating minimal but acceptable lattice distortion that could still lead to a solid solution formation. The valence electron concentration (VEC) of 9.081 is conducive for the formation of desirable phases, although the exact preferred phase (FCC, BCC, or HCP) is not specified, general VEC guidance suggests a structure that would support a stable solid solution. The mixing enthalpy of 10.7, while slightly positive, is low enough to suggest that with the chosen combination, the formation of a solid solution is still highly likely, minimalizing the risk of unwanted intermetallic compounds. However, the presence of Cu and Mn is not mentioned, which could impact corrosion resistance, conductivity, and phase stability. Also, the adherence to green manufacturing principles is explicitly evaluated in this data point. Overall, while some aspects slightly miss the optimal criteria, the chosen elemental composition and the reported physical properties suggest a high likelihood of forming a stable, high-performance HEA, pending further experimental validation.

② **Al28Co11Cr11Fe11Ni39**: The elemental selection follows the RULE by incorporating components (Al, Co, Cr, Fe, Ni) known for their compatibility in forming stable high-entropy alloys, promoting a single-phase solid solution. The atomic size difference of 5.169% falls within the optimal range (5%-15%), supporting solid solution formation with minimal lattice distortion, aligning with the atomic size difference RULE for stable HEAs. The valence electron concentration (VEC) of 10.07 suggests the alloy might favor the formation of desired phases (FCC, BCC), abiding by the VEC RULE guiding phase stability in HEAs. However, the mixing enthalpy of 16.372, although low, is positive, slightly deviating from the optimal RULE criteria favoring negative or very low positive values to minimize intermetallic compound formation, potentially affecting phase stability and mechanical properties. While this composition adheres mostly to the RULE criteria for high entropy alloys, the slight deviation in mixing enthalpy lowers its suitability score for experimental validation.

③ **Al14Co19Cr19Fe19Ni19Mn9**: The formula Al14Co19Cr19Fe19Ni19Mn9 possesses a composition leaning towards the commonly used elements in EHEAs, such as Al, Co, Cr, Fe, and Ni, promoting stable high-entropy mixtures. The atomic size deviation falls within the optimal range (4.19%), enhancing solid solution stability with minimal lattice distortion. The valence electron concentration (VEC) of 8.808 is indicative of a structure that could favor desired phase formations such as FCC or BCC, aligning with the VEC rule. The positive mixing enthalpy (10.685) suggests some tendency towards compound formation, which is generally unfavorable but can be mitigated by the chosen element mix. The presence of Mn, despite its benefits in altering fluidity and solidification, risks phase instability and casting defects. This, combined with the positive mixing enthalpy slightly deviating from ideal conditions, leads to reducing the score. However, the composition largely adheres to the principles of elemental selection, atomic size difference, and valence

electron concentration, demonstrating a high relevance for HEA research but acknowledging room for improvement in mixing enthalpy and Mn-related risks.

7 Fabrication Methods and Microstructural Changes of $Al_{14}(CoCrFe)_{19}Ni_{29}$ EHEA Under Tension

Alloy ingots with a nominal composition of $Al_{14}(CoCrFe)_{19}Ni_{29}$ were prepared by arc-melting in a high-purity argon atmosphere, using raw materials with purities exceeding 99.9 wt.%. The molten alloy was casted into a copper mold and remelted seven times to ensure chemical homogeneity. Dog-bone shaped tensile specimens with a scale length of 14 mm, a width of 4 mm, and a thickness of 1.5 mm were cut from the as-cast ingot via electric discharging machine. The tensile specimens were then polished using silicon carbide paper up to 2000 grit, and subjected to quasi-static uniaxial tensile testing on a universal testing machine at a strain rate of $10^{-3}s^{-1}$. Three measurements were taken for each sample and the average value was obtained.

Figure S6 provides a detailed microstructural analysis of the EHEA, $Al_{14}(CoCrFe)_{19}Ni_{29}$, with a specific focus on the B2 phase near the FCC/B2 interface. The Figure S6 highlights the complex interplay between the soft FCC and hard B2 phases, demonstrating how the GNDs are accommodated within the microstructure and contribute to the back-stress hardening phenomenon observed in the material.

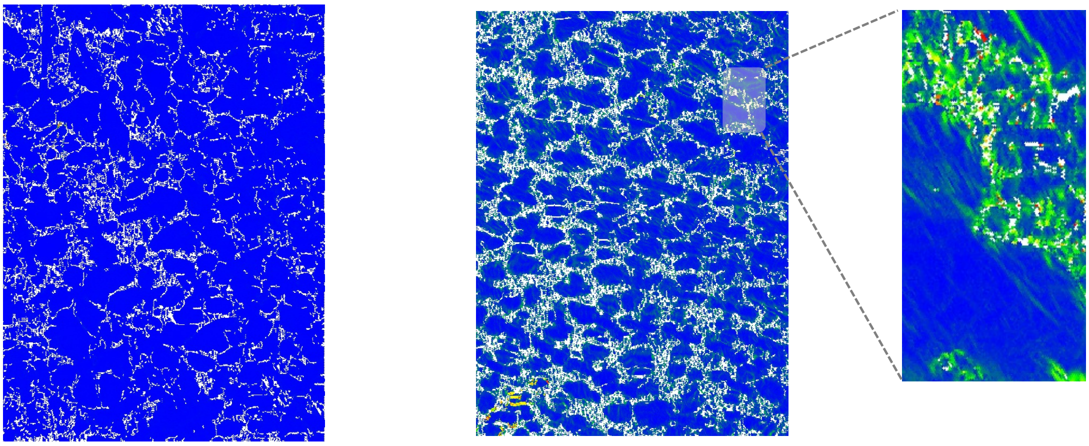

(a) before tension

(b) after tension

Fig. S6: The GND Density ($10^{12}/m^2$) of new-design HEA.

References

1. C. Wen, Y. Zhang, C. Wang, D. Xue, Y. Bai, S. Antonov, L. Dai, T. Lookman, and Y. Su, "Machine learning assisted design of high entropy alloys with desired property," *Acta Materialia*, vol. 170, pp. 109–117, 2019.

2. H. Zhuang, "From evidence to new high-entropy alloys," *Nature Computational Science*, vol. 1, no. 7, pp. 458–459, 2021.

3. R. Feng, C. Zhang, M. C. Gao, Z. Pei, F. Zhang, Y. Chen, D. Ma, K. An, J. D. Poplawsky, L. Ouyang *et al.*, "High-throughput design of high-performance lightweight high-entropy alloys," *Nature Communications*, vol. 12, no. 1, p. 4329, 2021.

4. C. Yang, C. Ren, Y. Jia, G. Wang, M. Li, and W. Lu, “A machine learning-based alloy design system to facilitate the rational design of high entropy alloys with enhanced hardness,” *Acta Materialia*, vol. 222, p. 117431, 2022.

5. H. Yamada, C. Liu, S. Wu, Y. Koyama, S. Ju, J. Shiomi, J. Morikawa, and R. Yoshida, “Predicting materials properties with little data using shotgun transfer learning,” *ACS central science*, vol. 5, no. 10, pp. 1717–1730, 2019.

6. S. Wu, Y. Kondo, M.-a. Kakimoto, B. Yang, H. Yamada, I. Kuwajima, G. Lambard, K. Hongo, Y. Xu, J. Shiomi *et al.*, “Machine-learning-assisted discovery of polymers with high thermal conductivity using a molecular design algorithm,” *Npj Computational Materials*, vol. 5, no. 1, p. 66, 2019.

7. D. Jha, K. Choudhary, F. Tavazza, W.-k. Liao, A. Choudhary, C. Campbell, and A. Agrawal, “Enhancing materials property prediction by leveraging computational and experimental data using deep transfer learning,” *Nature communications*, vol. 10, no. 1, p. 5316, 2019.

8. X. Li, Y. Dan, R. Dong, Z. Cao, C. Niu, Y. Song, S. Li, and J. Hu, “Computational screening of new perovskite materials using transfer learning and deep learning,” *Applied Sciences*, vol. 9, no. 24, p. 5510, 2019.

9. S. J. Pan, I. W. Tsang, J. T. Kwok, and Q. Yang, “Domain adaptation via transfer component analysis,” *IEEE transactions on neural networks*, vol. 22, no. 2, pp. 199–210, 2010.

10. E. Tzeng, J. Hoffman, N. Zhang, K. Saenko, and T. Darrell, “Deep domain confusion: Maximizing for domain invariance,” *arXiv preprint arXiv:1412.3474*, 2014.

11. M. Long, Y. Cao, J. Wang, and M. I. Jordan, “Learning transferable features with deep adaptation networks,” *arXiv: Learning*, 2015.

12. Y. Ganin and V. Lempitsky, “Unsupervised domain adaptation by backpropagation,” in *International conference on machine learning.* PMLR, 2015, pp. 1180–1189.

13. S. Sankaranarayanan, Y. Balaji, C. D. Castillo, and R. Chellappa, “Generate to adapt: Aligning domains using generative adversarial networks,” *arXiv: Computer Vision and Pattern Recognition*, 2017.

14. M. Long, Z. Cao, J. Wang, and M. I. Jordan, “Conditional adversarial domain adaptation,” *Advances in neural information processing systems*, vol. 31, 2018.

15. Z. Jiang, Y. Li, C. Yang, P. Gao, Y. Wang, Y. Tai, and C. Wang, “Prototypical contrast adaptation for domain adaptive semantic segmentation,” in *Computer Vision–ECCV 2022: 17th European Conference, Tel Aviv, Israel, October 23–27, 2022, Proceedings, Part XXXIV.* Springer, 2022, pp. 36–54.

16. Y. Wang, C. Wang, H. Xue, and S. Chen, “Self-corrected unsupervised domain adaptation,” *Frontiers of Computer Science*, vol. 16, no. 5, p. 165323, 2022.

17. E. DIN, “18265: Metallische werkstoffe-umwertung von härtewerten,” *Ausg. Febr*, 2004.

18. Yoshida-lab, “Xenonpy,” https://xenonpy.readthedocs.io, 2019.

19. Y. Yu, X. Wu, and Q. Qian, “Better utilization of materials’ compositions for predicting their properties: Material composition visualization network,” *Engineering Applications of Artificial Intelligence*, vol. 117, p. 105539, 2023.

20. I. Goodfellow, J. Pouget-Abadie, M. Mirza, B. Xu, D. Warde-Farley, S. Ozair, A. Courville, and Y. Bengio, “Generative adversarial nets,” *Journal of Japan Society for Fuzzy Theory and Intelligent Informatics*, 2014.

21. A. Odena, C. Olah, and J. Shlens, “Conditional image synthesis with auxiliary classifier gans,” *arXiv: Machine Learning*, 2016.

22. B. Sun and K. Saenko, “Deep coral: Correlation alignment for deep domain adaptation,” *Cornell University - arXiv*, 2016.

23. Y. Ganin and V. Lempitsky, “Unsupervised domain adaptation by backpropagation,” *International Conference on Machine Learning*, 2015.

24. Y.-H. Pao and Y. Takefuji, “Functional-link net computing: theory, system architecture, and functionalities,” *IEEE Computer*, 1992.

25. Q. Shi, R. Katuwal, P. N. Suganthan, and M. Tanveer, “Random vector functional link neural network based ensemble deep learning,” *Pattern Recognition*, 2021.

26. L. Van der Maaten and G. Hinton, “Visualizing data using t-sne.” *Journal of machine learning research*, vol. 9, no. 11, 2008.

27. H. Zhang, Y. Yang, L. Liu, C. Chen, T. Wang, R. Wei, T. Zhang, Y. Dong, and F. Li, “A novel feconicr0. 2si0. 2 high entropy alloy with an excellent balance of mechanical and soft magnetic properties,” *Journal of Magnetism and Magnetic Materials*, vol. 478, pp. 116–121, 2019.

28. M. Grant, M. R. Kunz, K. Iyer, L. I. Held, T. Tasdizen, J. A. Aguiar, and P. P. Dholabhai, “Integrating atomistic simulations and machine learning to design multi-principal element alloys with superior elastic modulus,” *Journal of Materials Research*, vol. 37, no. 8, pp. 1497–1512, 2022.

29. D. C. Ghosh and T. Chakraborty, “Gordy’s electrostatic scale of electronegativity revisited,” *Journal of Molecular Structure: THEOCHEM*, vol. 906, no. 1-3, pp. 87–93, 2009.

30. Y. Yusoff, M. S. Ngadiman, and A. M. Zain, “Overview of nsga-ii for optimizing machining process parameters,” *Procedia Engineering*, vol. 15, pp. 3978–3983, 2011.
